# Addressing Unmet Medical Needs in Drug Development: Assessment and Implications for Regulatory and Clinical Development Strategies

**DOI:** 10.3390/jmahp14010015

**Published:** 2026-03-09

**Authors:** Carla Domingo-Esteban, Inka Heikkinen, Nanco Hefting

**Affiliations:** 1 Department of Pharmacy, Faculty of Health and Medical Sciences, University of Copenhagen, 2100 Copenhagen, Denmark; carladomingo19@gmail.com; 2 H. Lundbeck A/S, 2500 Copenhagen, Denmark; inhi@lundbeck.com

**Keywords:** unmet medical need, drug development, regulatory science, health technology assessment, patient engagement, qualitative research

## Abstract

Unmet need is a core component of many Health Technology Assessment (HTA) processes at EU and national level. Most visibly, it is a core selection criterion for Joint Scientific Consultations (JSC) and Joint Clinical Assessment (JCA) for medical devices. This qualitative study explored how Unmet Medical Needs (UMNs) are understood and applied in drug development, with an emphasis on the European regulatory, HTA and access context, and examined their impact on regulatory and clinical development strategies. Twenty semi-structured interviews were conducted with representatives from regulatory authorities, HTA bodies, clinical development, industry, and patient insight roles. Data was analyzed using a thematic content approach combining deductive and inductive coding. Thematic analysis revealed general agreement on the importance of addressing UMNs, but also substantial variation in how they are defined and prioritized. Regulators often stressed disease severity and clinical evidence, while patients and clinicians emphasized quality of life. HTA representatives highlighted comparative benefit and long-term outcomes. These differing perspectives shaped how UMNs were integrated into development strategies, trial design, and regulatory planning. The findings indicate that clearer yet adaptable criteria could support earlier and more consistent alignment. Based on the analysis, a five-part roadmap to guide drug development is proposed, focusing on internal coordination, structured stakeholder engagement, collaboration between regulators and HTA bodies, adaptable definitions, and transparent decision-making. Together, these elements aim to support more systematic and predictable approaches to identifying and addressing unmet needs in drug development.

## 1. Introduction

Unmet medical need (UMN) often refers to situations where diagnostic, preventive, or therapeutic options are absent or inadequate, and it has become a central concept in pharmaceutical development and regulatory decision-making [1]. However, unmet needs may continue to exist despite approved products, due to issues related to inadequate response, patient preferences or side effects. Both regulatory and health technology assessment (HTA) dossiers, e.g., Electronic Common Technical Document (eCTD) and JCA template, require a description of UMN in the patient population the treatment is intended for, and some authorities tie prioritization (e.g., eligibility for a review pathway) or incentives (e.g., price algorithms) to the concept of UMN. In addition, UMN is a selection criterion for JSCs and medical devices selected for JCA [2]. Yet, absence of an aligned implementation of the concept across regulators, HTA bodies and stakeholder communities leads to inconsistent interpretations, shaping incentives and priorities unevenly [3].

Although UMN has guided innovation for years, its use continues to evolve in response to rare diseases and emerging health threats [4]. In 2023, the European Commission (EC) proposed a revision of pharmaceutical legislation, which among other objectives included formalizing UMN criteria to help steer development toward underserved conditions [5]. Concerns have been raised that some thresholds could be overly rigid and may overlook outcomes that matter to patients, reinforcing calls for more dynamic, patient-centered approaches [6,7,8]. Given the legislative interplay, changes to UMN definition in the Pharmaceutical Legislation would automatically apply to activities defined in the EU HTA Regulation as well. Therefore, changes to the definition will have relevance to both regulations but also at national level in EU countries.

The lack of a harmonized implementation of UMN has also created regulatory uncertainty and, in some cases, misaligned incentives [6]. A 2019 analysis showed that regulators, HTA agencies, industry, and patient groups apply criteria differently, weighing severity, availability of treatments, and population size according to their institutional perspectives [9]. This heterogeneity complicates prioritization, and makes it difficult to predict how UMN will be assessed [6]. If criteria are too narrow, areas with scientific progress that could translate into meaningful treatments for patients may be excluded, leading into incentives losing their effectiveness and discouraging investment [3].

Recognizing these challenges, many have proposed frameworks that preserve clarity while allowing flexibility [10]. The umbrella organization representing European rare disease patients, EURORDIS, has advocated a modular approach that distinguishes degrees of unmet need instead of relying on a binary definition [11]. The Belgian Knowledge Centre proposed a stringent scientific framework to identify and prioritize both patient and societal needs [12]. It has been recognized that flexibility is important in order to not exclude unmet needs unintentionally and allow case-by-case assessment, rather than automatically rate one disease higher than other [13,14].

One key element of EU HTA Regulation has been integrating patient voice better in the HTA decision-making. The patient voice has been central in highlighting quality of life and real-world treatment gaps, while HTA organizations traditionally focus more on comparative benefit and cost-effectiveness [8]. Early inclusion of patient perspectives, alongside clinician experience, can prevent inconsistencies between approvals and reimbursement outcomes [15,16]. Effective collaboration across regulators, HTA agencies, payers, clinicians, and patient representatives, is therefore essential [12]. Multi-stakeholder initiatives, such as the Netherlands Regulatory Science Network, stress inclusivity, transparency, and continuous revision for maintaining the relevance of UMN definitions [15]. Yet sustained dialogue remains necessary to align expectations [3].

UMN also influences developer decisions across the product lifecycle. In early research, it shapes target selection and the definition of intended patient populations. In clinical development, it guides the choice of endpoints, comparators, enrichment strategies, and acceptable uncertainty. During regulatory interactions, UMN considerations affect eligibility for expedited programs and the type of scientific advice sought, as well as evidence expectations [17]. In HTA and payer decision-making, it provides context for the decision at hand, the clinical relevance and may have a formal role in the decision frameworks in some countries [9]. After authorization, revisiting UMN supports post-marketing studies and real-world evidence (RWE) collection, keeping therapies aligned with evolving patient and system needs [18].

Within the European Union (EU), multiple regulatory instruments incorporate UMN; the Orphan Regulation (although it does not define UMN explicitly) and Conditional Marketing Authorization apply where no adequate method exists or a product is expected to provide significant therapeutic benefit [9,19]. The Priority Medicines (PRIME) scheme may connect to Accelerated Assessment for products expected to improve outcomes in high-need areas [19,20,21]. Together these mechanisms demonstrate how UMN has been embedded in EU regulatory policy for nearly two decades [1].

Beyond Europe, the challenges in defining and operationalizing UMN are evident in other regions. In the United States (US), the FDA’s expedited programs support development for serious conditions, yet debates persist about evidentiary standards when approvals rely on surrogate endpoints and how such decisions translate access [22,23]. Accelerated designations do not always lead to access if payer or HTA requirements diverge, especially around practical definitions and robustness of evidence [24].

These debates illustrate that drug development is a global effort, and inconsistencies in UMN applications create challenges for multinational R&D strategies. Improving alignment between regulators and payers internationally is therefore critical to ensure that expedited access pathways deliver both timely innovation and meaningful patient benefit [25,26,27].

The purpose of this study is to examine how UMN is defined and applied in drug development with a focus on the European regulatory, HTA and access context; it explores how stakeholder interpretations shape clinical and regulatory strategies. The aim is to propose elements for a roadmap that would enable earlier and more consistent identification of unmet needs, supporting predictable and patient-centered innovation.

## 2. Materials and Methods

### 2.1. Research Approach

Given the exploratory aim of the study to understand how stakeholders define and apply the concept of UMN, a qualitative design with semi-structured interviews was chosen. This approach is well suited for addressing “how” and “why” questions and for examining the reasoning behind stakeholder perspectives [28].

To support reliability and validity, interviews followed a common guide, they were audio-recorded and transcribed, and transcripts were reviewed multiple times. Triangulation with public sources such as regulatory and policy statements helped identify themes, confirm patterns, and highlight contradictions [29]. Researcher reflexivity was maintained throughout the study to minimize bias and strengthen transparency [30]. Given the industry-academic collaboration context, particular attention was paid to potential power dynamics and organizational alignment during data collection and analysis. Participants were informed about the academic and exploratory nature of the research and assured that responses would be anonymized and reported without attribution to specific individuals or organizations. Analytical decisions were regularly discussed under academic supervision to enhance interpretative transparency and mitigate potential social desirability effects.

### 2.2. Semi-Structured Interviews

Twenty semi-structured interviews were conducted in March–April 2025, each lasting 45–60 min. They took place in person when feasible or online via Microsoft Teams. Sessions opened with a brief project introduction, invited participants’ own UMN definitions, then presented the EC definition for comparison, and closed with open questions. All but one interview were audio-recorded; in the exceptional case, detailed notes were taken due to agency policy. Transcripts were generated using Microsoft Word’s transcription tool.

### 2.3. Sampling Strategy

Participants were selected using purposive sampling, with elements of convenience sampling [28,31]. The aim was to capture diversity across key stakeholder groups, including regulators, HTA and market access specialists, clinical development professionals, industry representatives, and patient insights experts, rather than numerical balance. Eligible participants were required to hold professional roles and relevant experience in regulatory, policy or strategic discussions related to UMNs. Both internal stakeholders and external informants were included, using professional networks and targeted outreach. Potential participants were identified based on publicly available professional roles, institutional affiliations, and demonstrated involvement in regulatory or policy discussions related to UMNs.

In total, 33 individuals were contacted, of whom 20 (60.6%) participated. Recruitment aimed to ensure inclusion of all predefined stakeholder categories and diversity across public and private sector perspectives. Non-participation was primarily due to limited availability within the study timeframe.

The study was conducted in accordance with University of Copenhagen guidelines for student research projects involving human participants and complied with the Danish Act on Research Ethics Review of Health Research. All procedures complied with the General Data Protection Regulation, GDPR, Regulation (EU) 2016/679. Participants were informed about the purpose of the study, the voluntary nature of participation, how their data would be processed and stored, and their right to withdraw prior to publication. Oral informed consent was obtained and recorded at the beginning of each interview. Data were anonymized, securely stored, and deleted after completion of the project in accordance with university data management requirements.

### 2.4. Interview Guide

The guide reflected the research objectives while allowing flexibility for open discussion [32]. A semi-structured format ensured consistency across conversations but allowed the interviewer to adapt follow-up questions and explore new themes when relevant [33].

Prior to data collection, the guide was piloted with eight colleagues representing the main stakeholder groups included in the study (three clinical experts, two HTA/market access professionals, two regulatory experts, and one patient-insight representative) to assess clarity, relevance, and logical flow. These pilot interviews were conducted to test comprehensibility and question sequencing and were not included in the final analytical dataset. Based on feedback, minor revisions were made to refine wording and improve the structure of the interview. Additional questions were introduced iteratively as recurrent topics emerged [34]. The final version of the semi-structured interview is provided in Supplementary File S1.

### 2.5. Data Analysis

A thematic content analysis was conducted, combining a deductive coding framework with inductive exploration [32,35]. An initial codebook was developed based on research questions, the literature, and the interview guide. This codebook defined the preliminary analytical categories that guided the first, deductive stage of coding.

Coding was conducted by the first author using NVivo 15 software. The coding framework was iteratively refined as new themes emerged during analysis. To enhance analytical rigor, coding decisions and emerging themes were regularly discussed under academic supervision to ensure conceptual consistency and transparency. Any interpretative uncertainties were resolved through discussion until agreement was reached. The final coding framework, including code definitions, is provided in Supplementary File S2.

The transcripts were then coded in NVivo in two stages: first, applying the predefined codes; second, conducting an inductive review to capture unanticipated but relevant themes [36]. Thematic saturation was considered reached when successive interviews did not generate new codes or materially alter the existing thematic structure [37]. Reliability was reinforced by double-checking all transcripts and checking codes across groups. Cross-case analysis compared interpretations by professional role to examine differences across contexts [34,38]. A summary of the anonymized key themes and insights derived from the interviews is provided in Supplementary File S3.

## 3. Results

Findings are presented thematically. Participants represented clinical experts (CE), HTA/market access (HTA/MA) professionals, regulatory experts (RE), and patient-insight (PI) roles. Quotations are labeled by stakeholder type and region.

### 3.1. How Stakeholders Define UMNs

#### 3.1.1. Criteria Emphasized

Across groups, UMN was understood as going beyond the absence of treatments, encompassing residual burden with existing options, poor tolerability, and limited impact on patient-relevant outcomes. Clinicians highlighted the disconnect between trial endpoints and daily function: “If you start off with 16 migraine days per month, that (50% reduction) still leaves you with two migraines a week, which is not a sufficient target” (CE, EU). Patient-insight specialists stressed social and emotional impact: “Patients frame need in terms of how a disease shapes the ability to live normally, including emotional and social life” (PI, EU). Regulators and HTA experts converged on the need for measurable evidence of benefit; convenience or preference alone was viewed as insufficient unless linked to outcomes. Overall, severity, treatment inadequacy, and patient-relevant impact were prioritized, though weighted differently across groups.

#### 3.1.2. Application Differs by Role

Within a pharmaceutical company, teams described UMN framing as evolving with the evidence and the treatment landscape over time. Regulators cautioned that a company’s evolving narrative about unmet need carries little weight unless supported by new evidence; as one put it, “Claiming UMNs is not enough; regulators make independent judgments based on data and context” (RE, UK). HTA voices favored population-level relevance and structured criteria over company-specific framings, underscoring tensions between feasibility (clinicians), consistency and evidentiary thresholds (regulators/HTA), and lived experience (patients). These role-linked lenses complicate early alignment on UMN and affect how programs are designed.

#### 3.1.3. Flexibility vs. Clarity

Most interviewees favored clearer yet flexible criteria. Clinical and regulatory experts cautioned that rigid definitions could miss needs in rare, heterogeneous, or progressive conditions. Others warned that overly broad definitions invite opportunism: “Looser definitions can lead to opportunism; more grounded criteria could focus efforts where need is truly urgent” (CE, US). HTA participants suggested graded frameworks or scoring systems that can be updated as evidence and practice evolve, while patient-insight specialists emphasized that perceptions of “unmet” change as expectations and standards shift. The consensus favored a stable framework with room for justified exceptions.

### 3.2. How UMN Perceptions Shape Development

#### 3.2.1. Early Strategy and Regulatory Designations

Within a pharmaceutical company, teams reported that uncertainty about endpoints and review expectations leads to de-prioritizing otherwise promising programs. Designations (e.g., PRIME/Breakthrough) were viewed as supportive signals but not guarantees, with participants emphasizing the importance of early regulatory dialogue “Early advice is essential for limited-evidence pathways” (RE, UK). Market-access interviewees described frequent disconnects between programs optimized for authorization and those that later meet HTA expectations for comparative, decision-relevant evidence.

#### 3.2.2. Aligning Regulatory and HTA Expectations

A recurring pain point was dual-aim trial design: satisfying authorization and reimbursement in one plan. HTA specialists highlighted that added value must be demonstrated, not inferred from novelty or convenience: “You can be in a space with no labelled treatment, and HTA still expects you to show you’re better than what’s being done now” (MA, EU). Participants noted country-specific requirements make alignment even harder. Patient-insight contributors observed that endpoints often miss what matters most to patients, undermining both relevance and recruitment.

#### 3.2.3. Feasibility as a Decision Filter

Programs were often stopped early due to scientific, operational, or regulatory feasibility, even where UMN is clear. “Scientific feasibility acts as a decision filter; programs are dropped if risk looks too high, even with unmet need” (CE, EU). Patient representatives argued that trials grounded in meaningful patient outcomes can improve engagement and feasibility, but limited validated measures and resistance to change restrict adoption. HTA and regulatory stakeholders noted that feasibility requires methods and endpoints acceptable to regulators and payers, not just convincing to sponsors. Some cited pilots like Belgium’s UMN NEED program, which enables earlier access when predefined needs are met [12].

#### 3.2.4. HTA Influence on Evidence Generation

Interviewees widely agreed HTA expectations increasingly shape development. Failure to plan for appropriate comparators, validated endpoints, and credible real-world relevance often results in authorization without reimbursement. “Think early about the comparator, if it’s not right for HTA, you’ve already lost part of the value argument” (MA, EU). Patient-insight specialists cautioned that conventional metrics can under-represent cognition, independence, or fatigue, potentially undervaluing benefits that matter to patients.

#### 3.2.5. UMNs Considerations in Drug Development

Stakeholders converged that identifying UMN early increases the odds of success: “Programs are only initiated if a clear unmet need is visible from the outset” (CE, EU). Regulators underscored that expedited pathways demand evidence of genuine need, not just strategic claims. Patient-insight roles advocated involving patients even at first-in-human stages to ensure endpoints and tolerability thresholds reflect real-world priorities. The shared message was the importance of cross-functional alignment on UMN before pivotal design choices.

### 3.3. Stakeholder Input and RWE

#### 3.3.1. Weight and Timing of Stakeholder Voices

Clinicians were seen as essential for feasibility and endpoint selection but may not fully capture lived burden in chronic or rare diseases: “Companies should independently [of KOLs] validate patient-centered priorities” (CE, US). Patient input is expanding and, when gathered early, is beginning to shape design: “We now interview patients as early as Phase 1 to clarify what ‘meaningful change’ really means for patients*”* (PI, EU). Regulators and HTA bodies recognized formal mechanisms for patient input but indicated that such input influences decisions only when linked to measurable benefit. Overall, engagement is improving but remains uneven and often late.

#### 3.3.2. Use of RWE to Justify UMNs

Stakeholders viewed RWE (patient registries, electronic health records) as useful to characterize unmet need, especially where Randomized Clinical Trials (RCTs) are impractical [39]. Participants noted that RWE captures daily functioning, long-term fluctuation, and service use not seen in trials; qualitative inputs can contextualize these signals. Regulators and HTA bodies emphasized that RWE must be methodologically rigorous, relevant to local care, and aligned with comparative questions. It is usually secondary to randomized evidence unless designed for decision-making, with greatest impact in evidence-scarce settings. Inconsistent planning, however, continues to limit its potential.

#### 3.3.3. Challenges in Incorporating Stakeholder Insights

Participants described a lack of clear responsibility for using patient input, limited tools to integrate it with clinical and regulatory priorities, and uncertainty about how to balance conflicting perspectives. While consultation mechanisms exist, they rarely alter the core evidentiary requirements. Concerns were raised about representativeness, with highly engaged ‘super patients’ sometimes dominating discussions, and about late engagement: “Internal pushback, resource concerns, and uncertainty about outcomes delay patient input until it can no longer guide strategy” (PI, EU). Many argued for earlier and more structured channels to link stakeholder insights directly to study decisions.

### 3.4. Regulatory Alignment and Predictability

#### 3.4.1. Portfolio Impact of Regulatory Definitions

While any development activity usually starts with assessing whether patient need exists, designations and UMN incentives support strategic framing but rarely drive portfolio selection, where scientific maturity, organizational fit, and risk remain the primary drivers. “Regulatory definitions rarely drive portfolio decisions directly; business factors override*”* (RE/MA, EU). Inconsistent application of UMN concepts across agencies complicates global planning, as recognition in one region may hold limited value elsewhere without robust evidence. HTA and patient-insight participants emphasized that national interpretation of need contributes to unequal access across Europe.

#### 3.4.2. Divergent Agency Requirements

Stakeholders noted that FDA pathways allow earlier and more adaptive development (e.g., rolling submissions), especially in rare disease, whereas EMA more often expects robust comparative data and endpoints aligned with HTA use. “EMA often requires head-to-head or active comparator trials; unlike FDA where such designs are often optional” (RE, EU). A former US regulator added that “FDA allows earlier engagements and faster timelines (e.g., rolling submissions), creating incentive to prioritize US development.” (RE, US). Participants linked these differences with later EU submissions and regional access gaps.

#### 3.4.3. Clarity, Consistency, and Room for Adaptation

Participants supported flexibility where standard designs are infeasible, paired with transparent boundaries: “Flexibility shouldn’t mean inconsistency, there needs to be a clear framework for when it is justified” (RE, UK). Even when regulators accept non-standard evidence, HTA bodies may still require comparative outcomes, creating an approval-access gap. Many interviewees pressed for earlier scientific dialogue and clearer reasoning to reduce avoidable uncertainty rather than lower standards.

### 3.5. Systematic Identification and Coordinated Development

#### 3.5.1. Early Identification Using Real World Data (RWD), Registries, and Clinician Input

Most participants favored identifying UMNs before formal development decisions. They described preclinical boards and cross-functional reviews, yet the integration of RWD, registry data, and structured clinician/patient input was inconsistent. Patient-insight and regulatory stakeholders supported early use of historical and real-world data to sharpen population definitions and endpoints, with caution about data quality and healthcare context. HTA voices called for practical tools (e.g., graded need frameworks) to align clinical, system, and patient needs at program start.

#### 3.5.2. Cross-Functional Collaboration

Interviewees agreed that earlier, more structured collaboration among research, clinical, regulatory, market access, and patient-insight teams would reduce misalignment. In practice, the extent to which patient input is used often depends on organizational culture and individual initiative rather than established procedures. “Despite acknowledging patient relevance, no structured channel exists to incorporate their input into research prioritization” (CE, EU). Externally, joint scientific consultation (JSC) pathways are available but underused, with sponsors citing unclear value proposition and timing constraints as common barriers.

#### 3.5.3. Strategic Approaches to Improve Predictability, Enhance Regulatory Alignment, and Expedite Development

Participants highlighted several approaches to improve predictability and alignment in development planning. These included introducing patient-relevant endpoints earlier (with validation plans), planning fit-for-purpose RWE, and treating HTA advice as design input rather than a late check. Regulators supported adaptive designs when justified. Organizational barriers, reluctance to take risks, resource concerns, and uncertainty about impact were seen as the main reasons patient input arrives late. HTA participants emphasized early comparator and endpoint choices as the most frequent avoidable gaps.

### 3.6. Where Stakeholders See Room for Change

#### 3.6.1. Suggestions for Improving Regulatory Frameworks and Classification

Stakeholders described current UMN frameworks [6] as fragmented and sometimes narrow, missing chronic burdens or non-drug solutions. Several called for transparent models that distinguish degrees of need while remaining adaptable over time. “Rigid symptom-based endpoints can dilute need relevance. Sometimes we just focus on other companies’ endpoints, just because they are doing it, and we miss the basics” (CE, EU). HTA interviewees warned that meeting authorization endpoints alone rarely demonstrates added value at the national level; broader tools are needed to capture system-level and long-term impact.

#### 3.6.2. Incentives and Policy

Views on EU Regulatory Data Protection (RDP) provision linked to the UMN framework [5] were mixed. Some participants questioned whether small extensions in exclusivity would influence high-risk investment decisions, especially if they are not aligned with HTA and payer expectations: “Six-month exclusivity extensions are unlikely to change company behavior” (HTA, EU). Many argued instead for incentives that directly support evidence generation relevant to access, such as head-to-head data, validated patient-reported outcomes, or post-authorization commitments tied to coverage, and for stronger links between incentive eligibility and real-world outcomes.

#### 3.6.3. Early Engagement with Regulators, HTA, and Patients

There was broad support for earlier, planned engagement to align on comparators, endpoints, and acceptable uncertainty, ideally before trial design begins: “Discussions on endpoints and comparators need to happen preclinically or at early design” (MA, EU). Patients were seen as critical to shaping meaningful endpoints and tolerability thresholds, but their input often arrives after key decisions, limiting its impact. Stakeholders urged building these exchanges into governance and timelines rather than treating them as optional.

#### 3.6.4. What Companies Could Change

Participants suggested four shifts: (i) start UMN articulation before asset selection and revisit it as evidence and landscapes change; (ii) make early patient engagement standard, with clear pathways to translate insights into endpoints and study design; (iii) treat HTA advice as central to pivotal planning; and (iv) design RWE programs prospectively to answer comparative and context-specific questions. As one regulator noted: “Early-stage development allows flexibility for innovation, including defining UMNs with patients and clinicians” (RE, UK). An HTA interviewee summarized the dual requirement: “HTA and regulatory have different data needs; development must satisfy both” (HTA, EU).

## 4. Discussion

### 4.1. Gaps and Overlaps in Operationalization of the Framework

UMN is central to drug development, regulation, and access, yet its operationalization remains inconsistent across stakeholders [9,10]. While severity, treatment gaps, and patient burden are broadly acknowledged, each group weighs these elements differently. Patient representatives emphasize daily burden, function, and social participation, aspects often excluded from formal frameworks [8]. Regulators apply structured criteria such as absence of satisfactory treatment and expedited programs like PRIME in Europe and Breakthrough Therapy in the United States [10,40]. These frameworks provide clarity but are criticized for limited transparency and inconsistent application.

HTA bodies add a comparative perspective, providing associated incentives only when products demonstrate added benefit. Gains in convenience or tolerability are insufficient unless tied to measurable outcomes, as highlighted by agencies such as IQWiG and EUnetHTA, which prioritize robust endpoints and head-to-head trials [41,42]. Consequently, medicines approved due to high UMN (usually through conditional approval or accelerated assessment) may face reimbursement delays if provided evidence does not meet the HTA requirements [43].

Clinicians expressed frustration with these divergences. Broader frameworks can capture functional or emotional burdens but are harder to operationalize, while narrow definitions provide consistency yet risk excluding underserved groups [44]. EURORDIS has warned that restrictive criteria often overlook rare and ultra-rare diseases [11]. Comparative analyses confirm that no single definition can satisfy all perspectives, though partial alignment is possible [8,10].

Differences between agencies also shape strategy. The FDA is seen as more flexible and proactive, particularly in high-need areas, while the EMA is viewed as predictable but more conservative. Many stakeholders therefore support layered definitions: a fixed core, such as absence of satisfactory treatment, complemented by contextual factors like disease fluctuation, comorbidities, or subgroup heterogeneity.

A transparent and flexible approach, grounded in multiple evidence types, was seen as essential to reduce misalignment and improve planning coherence.

### 4.2. How Definitions Translate into Strategy

How UMN is defined in early planning affects portfolio choices, evidence strategies, and trial design. Pharmaceutical company teams often adopt broader framings at the outset, narrowing them later to fit regulatory or HTA expectations. This flexibility helps manage uncertainty but complicates alignment across functions and with external decision-makers. Promising projects may be deprioritized when endpoints are unvalidated or comparators unclear. In these settings, the FDA’s greater openness to adaptive designs has been contrasted with the EMA’s and HTA bodies’ stronger insistence on comparative outcomes.

Regulatory approval does not guarantee access, since reimbursement decisions may be restricted or negative when trial designs neglect HTA standards [45]. Even if private markets provide some entry, this rarely translates into broad patient availability. Country-specific criteria add further uncertainty. Tools such as JSC could help address these gaps, yet their use remains limited, with barriers including low awareness, poor timing, and doubts about value. Empirical studies confirm that such advice supports convergence on endpoints but less so on comparators, which remain a key HTA demand [46]. The new EU HTA Regulation may improve consistency in early dialogue, though its practical impact remains unclear as implementation is still at an early stage [41,47].

In practice, feasibility, organizational fit, and market considerations often outweigh UMN-related incentives in portfolio selection. Greater clarity across regulators, HTA bodies, and companies from the outset could reduce costly redesigns and strengthen efficiency [8].

### 4.3. The Role of Stakeholder Engagement

Stakeholder engagement is recognized as essential, but in practice it remains inconsistent and often too late to influence strategy. EURORDIS has noted that much engagement occurs after decisions are already fixed, reducing it to a formality [11]. The FDA’s Patient Focused Drug Development program illustrates the potential of structured frameworks, but its impact depends on how systematically and early it is applied [48]. Patients, clinicians, and payers are increasingly consulted, yet their contributions rarely shape final decisions, as engagement often depends on individual initiative and limited mechanisms for integration across functions.

Representativeness is another concern. Engagement frequently relies on a small number of highly informed advocates, sometimes called “super patients”, whose perspectives may not reflect the wider community. In rare diseases, variability and fragmented advocacy make inclusivity even harder. Reviews confirm that engagement is concentrated at trial design rather than early R&D, limiting its influence [49,50].

When embedded earlier, engagement can bridge clinical relevance, patient experience, and regulatory standards, ensuring outcomes and tolerability thresholds are meaningful in practice. Unless systematically institutionalized, this potential is lost and engagement remains procedural rather than strategic. Case studies show that patient expert boards or similar mechanisms can embed perspectives directly into R&D planning [51]. Achieving this requires companies to build structured processes that translate engagement into strategic choices, making it both inclusive and impactful.

In addition to qualitative engagement, structured patient preference (PP) studies have been proposed as a formal approach to capture which treatment attributes and outcomes matter most to patients and how patients weigh potential benefits against harm. The IMI PREFER project has issued recommendations providing guidance on how to conduct, assess, and incorporate outcomes of patient preference studies in decision-making throughout the medical product life cycle, specifically in product development, regulatory decision-making/marketing authorization, and HTA or reimbursement contexts [52]. The recommendations further highlight the role of preference studies in informing the selection of patient-relevant endpoints and assessing the acceptability of benefit–risk trade-offs. Consistent with this, Janssens et al. report broad multi-stakeholder support for the use of patient preferences across the medical product lifecycle, while noting that uptake remains limited due to gaps in standardization and uncertainty regarding how preference evidence should be evaluated and integrated into regulatory and reimbursement decisions [53]. In the context of UMN, these approaches are particularly relevant, as they can help make patient-defined relevance more explicit when selecting endpoints and designing evidence generation strategies intended to inform downstream decision-making.

### 4.4. The Strategic Role of Industry in Defining and Supporting UMNs

Companies not only respond to UMNs but also play an active role in defining and prioritizing them. Internal framings often shift across development stages to align with regulatory or HTA goals. Planning tools such as product concept plans or unmet need templates exist but are inconsistently applied, with decisions frequently based on precedent or expert opinion rather than systematic assessment. This undermines alignment later in development. JSC could support convergence but are underused due to administrative burden and narrow technical scope [41,54].

Evidence generation is also shaped by how industry frames UMNs. RWE is increasingly recognized as essential to justify unmet need where RCTs are infeasible. Initiatives such as DARWIN EU aim to institutionalize RWD in regulatory decisions [55]. FDA guidance and recent approvals likewise signal greater acceptance when evidence is methodologically robust [56]. To maximize impact, such evidence must meet high methodological and ethical standards, with early planning, patient-relevant endpoints, and integration across functions [56,57].

By aligning internal tools, stakeholder engagement, and evidence strategies early, companies can promote a shared, patient-centered understanding of UMNs across the system. This early framing is strategic, as it shapes both regulatory feasibility and patient relevance.

### 4.5. Towards a Roadmap: Key Elements Identified

**Figure 1:**
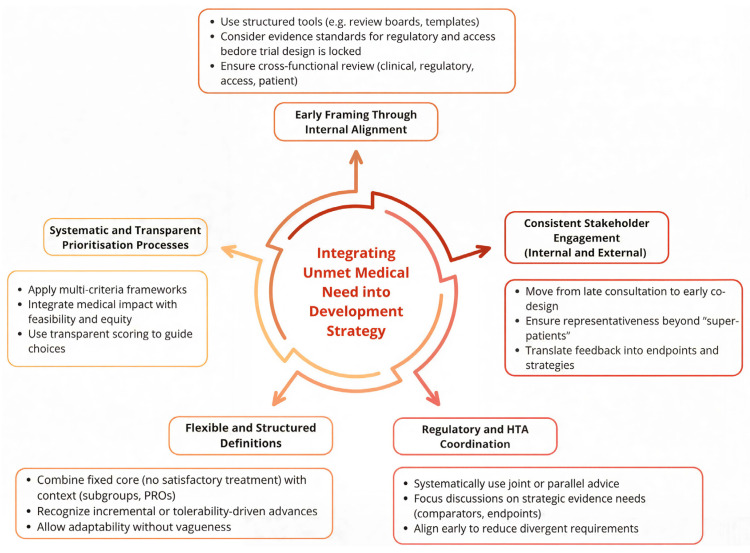
Roadmap for integrating unmet medical need into early development planning. The five interdependent elements reflect key themes identified through stakeholder interviews.

Five themes emerged as building blocks for a roadmap to improve how UMNs are defined and integrated: early internal alignment, consistent stakeholder engagement, coordination between regulatory and HTA expectations, flexible but structured definitions, and systematic prioritization (Figure 1).

Early framing through structured tools can prevent assumptions from becoming fixed too late, ensuring that unmet need is considered before design choices are locked in. Consistent engagement requires moving beyond late consultation toward co-design, with input that is representative and translated into trial endpoints and strategies [11,49]. Coordination between regulatory and HTA expectations depends on the systematic use of JSC as a strategic rather than purely technical tool [46]. Flexible but structured definitions can combine a fixed core with contextual dimensions such as subgroup variability or patient-reported outcomes [8,9,10].

Together these elements provide the basis for a roadmap that embeds UMN considerations from the outset. The aim is not rigidity but greater consistency, ensuring that development strategies reflect both regulatory feasibility and patient experience.

This study has several strengths. First, it included a diverse group of stakeholders across regulatory, HTA, clinical development, industry, and patient insight roles, enabling the exploration of converging and diverging perspectives within the drug development ecosystem. The inclusion of both public and private sector informants allowed for cross-contextual comparison and enriched the analysis. Second, a systematic qualitative methodology was applied, combining deductive and inductive coding, iterative refinement of the coding framework, and cross-case analysis. The use of semi-structured interviews enabled in-depth exploration while maintaining thematic consistency across participants.

However, several limitations should be acknowledged. The sampling strategy relied partly on professional networks, which may have introduced selection bias and limited inclusion of alternative or more critical viewpoints. Numerical representation across stakeholder categories was uneven due to availability constraints. The sample size (n = 20), while appropriate for qualitative thematic exploration, limits transferability of findings. In addition, direct patient representatives were not included; instead, patient perspectives were represented through patient insight professionals, which may not fully capture lived patient experience. As interviews involved professional stakeholders and were conducted within an industry-academic collaboration context, the possibility of social desirability bias cannot be fully excluded. These factors should be considered when interpreting the findings.

## 5. Conclusions

The findings underline that UMN is not only a technical concept but also a political one due to associated incentives (price premiums, regulatory flexibility, longer data protection). It is therefore important to reflect whose perspectives are included and whose standards are applied and focus on objective and inclusive assessment of the existing need. For the UMN framework to guide innovation effectively, it must be embedded early and applied consistently, balancing evidence requirements with patient experience. It should also provide predictability, ensuring that the UMN designation and related incentives remain stable throughout the product development lifecycle, since investment decisions occur much earlier than the UMN assessment, which takes place relatively late. Achieving this requires not only legislative reform but also organizational and cultural change in how companies and authorities interpret UMNs.

By clarifying definitions and aligning expectations earlier, drug development can become more predictable and responsive to patients, improving the translation of innovation into access.

## Data Availability

The qualitative interview transcripts generated and analyzed during the current study are not publicly available due to confidentiality and ethical restrictions related to participant consent. The datasets are therefore not readily available. De-identified summaries of key thematic insights supporting the reported results are included in the Supplementary Materials.

## Supplementary Materials

The following supporting information can be downloaded at: https://www.mdpi.com/article/10.3390/jmahp14010015/s1, Supplementary File S1: Summary of anonymized key themes and insights derived from the qualitative interviews. Supplementary File S2: Coding Framework; Supplementary File S3: Thematic summary of key ideas, grouped by stakeholder type.
