# Supplementary material for: Addressing Unmet Medical Needs in Drug Development: Assessment and Implications for Regulatory and Clinical Development Strategies
Source: J Mark Access Health Policy. 2026 Mar 9;14(1):15. doi: 10.3390/jmahp14010015 (PMC13027914; doi:10.3390/jmahp14010015)
Supplement: Supplementary file 1 [file jmahp-14-00015-s001.zip › Supplementary Materials/S1 - Interview guide.pdf]

Supplementary Material S1: Semi-structured Interview Guide

Research questions of the project: (info gathered from the interviews)

1- How do different stakeholders perceive and define UMNs in practice, and how does this influence drug development?

2- How can pharmaceutical companies incorporate patient and clinician insights into regulatory strategies to better define UMNs and align development plans with medical needs?

3- What key elements should a roadmap include to systematically and effectively identify and address UMNs, while ensuring regulatory and HTA alignment and enhancing predictability in drug development?

Introduction (common to all participants):

The interview lasts approximately one hour. There are no right or wrong answers; we are interested in your perspectives and experiences. Participation is voluntary and you may withdraw at any time without providing a reason. With your consent, the interview will be audio-recorded to ensure accurate capture of information. All responses will be anonymized and used for academic purposes only (master's thesis and potential scientific publication). No personally identifiable information will be included, and quotes will be reported in a way that is not attributable to you or your organisation. Participation involves no foreseeable risks. By agreeing to participate, you confirm that you have been informed about the purpose of the interview, how the data will be used, and your rights as a participant, and that you consent to participate and to audio recording.

Objective of the project (brief overview shared with participants):

This research explores how UMNs are understood and applied in drug development and decision-making across stakeholders. UMN is frequently referenced in regulatory and HTA contexts, yet definitions and operational criteria vary across stakeholder groups and jurisdictions. In the EU context, recent legislative proposals have further intensified the debate on how to define UMN while balancing clarity and flexibility. The objective of this study is to explore stakeholder perspectives on UMN and their implications for clinical and regulatory development strategies, including how patient and clinician perspectives are considered. The goal is to support more predictable and patient-relevant approaches to identifying and addressing unmet needs.

Interview format and structure:

Interviews followed a semi-structured format. A general interview guide was used across participants to ensure consistency and comparability, while allowing flexibility to explore emerging themes. Participants were first invited to share their own understanding and definition of UMN. Subsequently, the European Commission's proposed definition was introduced to prompt reflection and comparison. Interviews concluded with open-ended questions to allow additional perspectives not covered in the guide. Background information on each participant's professional role and institutional context was reviewed prior to interviews, and minor wording adaptations were made where needed to ensure relevance for the specific role without changing the thematic structure of the guide.

Semi-structured Interview Guide:

Patients representatives:
1. Understanding Unmet Medical Needs from a Patient Perspective<br>- How do you define an unmet medical need (UMN) from a patient perspective?<br>- What factors make a condition or situation truly “unmet” in your disease area?<br>- Are there needs that you believe are overlooked or insufficiently prioritized in drug development?<br>2. Impact of Unmet Needs on Patients<br>- Which aspects of the condition are not adequately addressed by current treatments?<br>- How do these gaps affect patients’ quality of life, daily functioning, and long-term well-being?<br>- Are there symptoms or outcomes that you feel are insufficiently considered in treatment development?<br>3. Patient Involvement in Drug Development and Regulatory Decision-Making<br>- How should patient perspectives be incorporated into drug development and regulatory processes?<br>- What role can patient input play in shaping clinical trial design and the definition of UMNs?<br>- How should patient-reported outcomes and real-world evidence contribute to identifying and addressing unmet need?<br>- What criteria should be prioritized when assessing whether a medical need is considered “unmet”?<br>4. EU Pharmaceutical Reform and the Definition of UMN<br>- The revised EU pharmaceutical legislation distinguishes between general UMNs and high unmet needs in orphan diseases. Do you consider this distinction meaningful from a patient perspective?<br>- Should UMN be treated as a binary category, or would a graded approach better reflect patient realities?<br>5. Access, Incentives, and Market Challenges<br>- What challenges have patients faced in accessing new treatments addressing UMNs?<br>- In your view, what changes could improve timely and equitable access?<br>- Are there systemic barriers, such as reimbursement, availability, or regulatory delays, that particularly affect patients in your disease area?<br>6. Future Improvements and Roadmap<br>- How can pharmaceutical companies and regulators involve patients earlier in identifying and addressing UMNs?<br>- What changes to regulatory frameworks could improve alignment between patient needs and development strategies?<br>- If you could propose one key improvement to how UMNs are identified and addressed in drug development, what would it be?

Regulatory/Policy Experts
1. Understanding and Defining UMNs in a Regulatory Context<br>- From a regulatory perspective, how is unmet medical need defined or applied in practice?<br>- What criteria are most important when determining whether a condition or product addresses an UMN?<br>- How do you balance the need to encourage innovation with the requirement for robust clinical evidence?<br>- Do you believe the current regulatory framework adequately captures all types of unmet need, or are there gaps?<br>2. Regulatory Role in Addressing UMNs<br>- How do you see the role of regulatory authorities such as the EMA or FDA in identifying and addressing UMNs during drug development?<br>- What challenges do regulators face when assessing whether a product truly addresses an unmet need?<br>- How does regulatory decision-making influence the prioritization of certain disease areas or conditions?<br>3. Integration of Patient and Clinician Perspectives<br>- How are patient and clinician perspectives incorporated into regulatory assessments of UMNs?<br>- Do current frameworks sufficiently integrate patient-reported outcomes and real-world evidence?<br>- What challenges arise when translating patient needs into regulatory criteria and decisions?<br>- In your view, are pharmaceutical companies effectively incorporating patient and clinician input into their UMN strategies? What improvements could strengthen this process?<br>4. EU Pharmaceutical Reform and UMN Classification<br>- The revised EU pharmaceutical legislation introduces distinctions between general UMNs and high unmet needs in orphan diseases.<br>- Do you consider this distinction to improve regulatory clarity, or does it introduce additional complexity?<br>- How might this distinction influence company behavior or development priorities?<br>- Do you expect the revised framework to enhance predictability for developers, and what potential unintended consequences should be considered?<br>5. Incentives and Regulatory Support<br>- Beyond financial incentives, what regulatory tools or mechanisms could better support the development of treatments addressing UMNs?<br>- How can approval pathways be optimized to accelerate access while maintaining standards of safety and efficacy?<br>- Are existing tools such as expedited pathways, conditional approvals, or scientific advice being used effectively in the UMN context?<br>6. Final Reflections<br>- If you could introduce one improvement to how UMNs are identified, assessed, or incentivized within the regulatory system, what would it be?<br>- What would be the main challenge in implementing such a change?<br>- How should regulatory frameworks evolve to remain relevant as scientific and technological advances reshape the landscape of unmet need?

Clinical Development Experts

1. Defining and Identifying UMNs in Clinical Development
- How do you define or operationalize unmet medical need when designing clinical development programs?
- What criteria are most important when assessing whether a condition or indication represents an UMN, for example severity, lack of effective treatments, patient burden, or long-term outcomes?
- At what stage in development do discussions about UMN typically begin, and how do they influence program prioritization?
- Do you see a gap between regulatory definitions of UMNs and what you observe in clinical practice? Are certain conditions under-recognized as UMNs from a regulatory perspective?
- How do such gaps affect trial design or overall development strategy?
2. Regulatory Alignment and Development Strategy
- How do regulatory expectations shape your approach to developing treatments for UMNs?
- How do you balance the urgency of addressing unmet need with requirements for robust clinical evidence?
- Do current regulatory frameworks provide sufficient clarity and predictability when planning development programs targeting UMNs?
- How do distinctions introduced in the revised EU legislation, such as general versus high UMNs, influence your development planning?
3. Integration of Patient and Clinician Perspectives
- How do you incorporate patient and clinician insights when identifying UMNs and selecting endpoints?
- What challenges arise when translating patient-relevant outcomes into measurable clinical endpoints?
- Do you believe current regulatory frameworks sufficiently accommodate patient-reported outcomes and patient-centered endpoints?
- How could earlier or more structured engagement improve alignment between clinical development and patient needs?
4. Role of Real-World Evidence
- How do you use, or plan to use, real-world evidence to support the identification or justification of an UMN in development programs?
- What challenges do you encounter in generating real-world evidence that aligns with regulatory and payer expectations?
- Do you believe regulators and HTA bodies adequately consider real-world data when assessing whether a treatment addresses an UMN?
- What improvements could strengthen the role of real-world evidence in this context?
5. Late-Stage Development and Market Access
- How do UMN considerations influence late-stage trial design, particularly regarding primary endpoints, comparators, and patient-reported outcomes?
- What challenges arise when trying to design trials that satisfy both regulatory approval and HTA or payer requirements?
- How do you manage the tension between demonstrating added value and accelerating access for patients with high unmet need?
6. Incentives and Future Evolution

- How do regulatory incentives, such as expedited pathways or data protection extensions, influence development strategies for UMNs?
- What additional regulatory tools or changes could better support clinical development in areas of high unmet need?
- If you could introduce one improvement to how UMNs are defined or operationalized in clinical development, what would it be?

Clinical development experts working in early research

1. Identifying Unmet Medical Needs in Early Research
- How do you define or conceptualize an unmet medical need when considering a new research project?
- What key factors influence your decision to prioritize a condition as addressing an UMN, such as disease severity, lack of effective treatments, disease progression, or population size?
- How do you assess whether a disease area qualifies as an UMN before initiating research?
- Are there specific tools or data sources you rely on, such as patient registries, epidemiological data, or real-world evidence?
- How do you balance scientific feasibility with the urgency of unmet need at this early stage?
2. Alignment with Regulatory and Industry Expectations
- To what extent do regulatory definitions of UMNs influence your research priorities? Do you find current definitions sufficiently clear to guide early-stage research decisions?
- What challenges arise when attempting to align exploratory research with existing regulatory frameworks for UMNs?
- How do patient perspectives and clinician input influence your research agenda?
- Can you provide an example where patient or clinician feedback led to a change in research focus or prioritization?
3. Scientific Feasibility and High Unmet Need
- How do you navigate situations where a disease represents a high unmet need but is scientifically complex or difficult to target?
- Have there been cases where a promising area of high unmet need was deprioritized due to feasibility constraints?
- How do you evaluate whether a potential therapeutic approach is realistically developable while still addressing urgent medical needs?
- How are emerging technologies, such as gene therapies or AI-driven discovery platforms, reshaping this balance between feasibility and unmet need?
- Do you believe current UMN definitions are flexible enough to accommodate scientific advances, or should they evolve as innovation progresses?
4. Collaboration and Translation into Development
- How do you collaborate with clinical development, regulatory, or market access teams during the early research phase to ensure future alignment around UMNs?
- Does early cross-functional engagement improve the transition from discovery to clinical development?
- If you could propose one improvement in how UMNs are identified and prioritized at the research stage, what would it be?

- How could such a change better align research priorities with patient needs and regulatory expectations?

Market access / Payer perspective

1. Defining and Prioritizing UMNs in Reimbursement Decisions
- From a market access or payer perspective, what key factors drive the identification and prioritization of unmet medical needs for reimbursement? For example, disease severity, availability of alternatives, patient burden, treatment gap, or cost-effectiveness.
- How do these factors influence decisions on whether a treatment addressing an UMN should be reimbursed?
- How do you assess whether a claimed UMN aligns with both regulatory definitions and payer or HTA expectations?
- Do discrepancies arise between regulatory recognition of UMN and reimbursement criteria, and how are these managed in practice?
2. Evidence Requirements and Value Demonstration
- What types of evidence are most critical when assessing the value of a therapy targeting an UMN?
- Are randomized clinical trials sufficient, or is there increasing reliance on real-world evidence and patient-reported outcomes to support reimbursement decisions?
- How do you balance clinical trial data with real-world evidence when evaluating UMN therapies?
- Can you provide an example where additional evidence, such as real-world data, influenced a reimbursement outcome?
3. Reimbursement Challenges and Alignment with Regulation
- What are the main obstacles to securing reimbursement for therapies addressing UMNs?
- How do affordability, budget impact, or uncertainty in evidence affect decisions, even when a clear unmet need exists?
- How are situations handled where there is high unmet need but limited cost-effectiveness under conventional thresholds?
- In what ways could regulatory approval processes be better aligned with payer or HTA requirements to facilitate timely access?
- Do you consider existing regulatory incentives, including mechanisms such as extended data protection, sufficient to encourage development in UMN areas from a payer perspective?
4. Collaboration and Future Policy Development
- What measures could improve collaboration between regulators, payers, and industry to strengthen recognition and prioritization of UMNs in access decisions?
- How might earlier engagement with payers during development improve alignment between approval and reimbursement outcomes?
- How should reimbursement systems evolve to better support therapies addressing UMNs, particularly innovative or rare disease treatments?
- What changes would you recommend to improve how UMNs are defined and prioritized in light of ongoing scientific and technological advances?
