# Supplementary material for: Addressing Unmet Medical Needs in Drug Development: Assessment and Implications for Regulatory and Clinical Development Strategies
Source: J Mark Access Health Policy. 2026 Mar 9;14(1):15. doi: 10.3390/jmahp14010015 (PMC13027914; doi:10.3390/jmahp14010015)
Supplement: Supplementary file 1 [file jmahp-14-00015-s001.zip › Supplementary Materials/S2 - Codes Interviews.pdf]

Supplementary Material S2- CODING FRAMEWORK

The coding framework combined predominantly deductive (a priori) codes with a limited number of inductively derived codes. The initial codebook was developed prior to analysis based on the research questions, literature review, and interview guide structure. During coding, a small number of additional codes were generated inductively to capture themes that were not fully represented within the predefined framework. These inductive codes were incorporated to ensure that emergent insights were not overlooked.

1. Definition & Interpretation of UMNs - How Stakeholders Define UMNs

- Definition: Statements describing how stakeholders conceptualize or define unmet medical needs (UMNs), including criteria and interpretative differences.

- Criteria emphasized
- Application differs by role
- Flexibility vs. Clarity in UMN Designation

2. How UMN perceptions shape development

- Definition: Statements describing how interpretations of UMNs influence strategic, clinical, or regulatory decisions during drug development.

- Early strategy and regulatory designations
- Aligning regulatory and HTA expectations
- Feasibility as a decision filter (inductive)
- HTA influence on evidence generation
- UMNs considerations in drug development

3. Stakeholder input and RWE

- Definition: References to stakeholder involvement and the use of real-world evidence in identifying or justifying UMNs.

- Weight and timing of stakeholder voices
- Use of RWE to justify UMNs
- Challenges in incorporating stakeholder insights

4. Regulatory alignment and predictability

- Definition: Statements relating to consistency, divergence, or uncertainty across regulatory bodies and HTA institutions.

- Portfolio impact of regulatory definitions (inductive)
- Divergent agency requirements
- Clarity, consistency, and room for adaptation

5. Systematic identification and coordinated development

- Definition: Statements describing structured approaches to identifying UMNs and improving coordination across functions.

- Early identification using Real World Data (RWD), registries, and clinician input
- Cross-functional collaboration
- Strategic approaches to improve predictability, enhance regulatory alignment, and expedite development

6. Where stakeholders see room for change

- Definition: Suggestions or proposals for improving UMN frameworks, incentives, or governance structures.

- Suggestions for improving regulatory frameworks and classification
- Incentives and policy
- Early engagement with regulators, HTA, and patients
- What companies could change
