# Supplementary material for: Addressing Unmet Medical Needs in Drug Development: Assessment and Implications for Regulatory and Clinical Development Strategies
Source: J Mark Access Health Policy. 2026 Mar 9;14(1):15. doi: 10.3390/jmahp14010015 (PMC13027914; doi:10.3390/jmahp14010015)
Supplement: Supplementary file 1 [file jmahp-14-00015-s001.zip › Supplementary Materials/S3 - Key ideas interviews.pdf]

Supplementary Material S3:Thematic Summary of Key Ideas, Grouped by Stakeholder Type

This section presents a thematic summary of the key ideas identified during the coding process, organized by stakeholder group. To ensure completeness and facilitate further analysis, I have included a list of individual code summaries developed throughout the coding phase. This serves as a supplementary check to capture any potentially overlooked insights.

The information is structured as follows:

Stakeholder Type (Internal / External) - Stakeholder Group - Summary of Key Ideas by Code

This organization allows for a clearer understanding of recurring themes and how they relate to the perspectives of different stakeholder categories.To optimize space and improve readability, the list is presented in two columns. Individual names have been removed to ensure anonymity and comply with ethical standards for data protection.

Note on color coding:

- Codes highlighted in **black** correspond to primary codes identified during the initial round of coding.
- Codes highlighted in **orange** indicate insights that were added or refined during the second reading of the data.

Summary of codes:

1. Definition and criteria for UMNs

1.1. Criteria for UMNs (severity, treatment gap, rarity…)

Clinical Stakeholders

Internal

Clinical professionals inside the company define UMNs through a pragmatic lens, balancing clinical outcomes, real-world complexity, and patient experience.

- UMNs are present even when approved treatments exist, if outcomes are poor or side effects are significant.
- Conditions that significantly disrupt daily life or functional capacity indicate ongoing unmet need.
- Mortality and morbidity matter, but clinicians emphasize the **gap between clinical response and patient functioning**, especially in chronic illness. *[Expanded via transcript]*
- **Treatment-related burden** — such as drug side effects or complexity of regimens — is perceived as a contributor to unmet need. *[From transcript]*
- Program initiation is often blocked unless the **UMN is precisely defined**, showing that strategic feasibility and need definition are tightly linked. *[From transcript]*

External

External clinicians and ex-regulators define UMNs with a sharper focus on therapeutic gaps and regulatory frameworks.

- UMNs are clearly present where **no effective treatments exist**, or where large groups don't respond to available options.

- Definitions of unmet need inside pharma companies are also shaped by **commercial filters** — unmet need must often align with expected market or regulatory return.*[Expanded via transcript]*
- Certain cases are **more "disputable" or context-sensitive**, such as where efficacy exists but is marginal, or only in some populations.
- Burden on **social functioning and employability** was mentioned as part of the unmet need definition.
- **Rare diseases** are seen as high unmet need zones, but also face data limitations and barriers to validation.

Regulatory Affairs (RA)

Internal

Internal RA experts emphasize **strategic and evidentiary consideration s** in defining UMNs:

- UMNs are not always binary; sometimes **partial efficacy, poor tolerability, or narrow population coverage** still qualify as unmet need.
- The **degree of unmet need** is a central factor in portfolio strategy — not just its presence.
- UMNs also include conditions that are **non-lethal but cause severe life limitations**, like functional dependence or constant care.
- To justify investment or development, the **need must be well-scoped and evidence-backed**.

External

- Formal frameworks like MHRA's **PIM** provide structured UMN criteria:

1. *Serious condition*
2. *Added benefit*
3. *Favorable risk-benefit*

- Even when treatments exist, UMNs can be justified based on **suboptimal response or tolerability**, especially in subgroups.
- Evaluation of UMNs is **multifactorial and contextual**: disease severity, patient population, current standard of care, and available alternatives.
- Regulatory definitions are **not universal**; authorities make **case-by-case assessments**.

HTA / Market Access

Internal

Stakeholders from Market Access, Global Brand Strategy, and Asset Planning offer a **strategic, system-level view** of UMNs:

- UMNs for HTA require more than regulatory approval — there must be **demonstrable added value**, in terms of health outcomes, costs, or quality of life.
- **Comparator choice is critical**: using outdated comparators might help approval but harms HTA acceptance in countries like Germany or France.
- Prioritization is necessary: **not all unmet needs are equal**, and HTA frames must assess impact and societal relevance.
- Market-facing functions expect products to **surpass standard of care**, not just be incrementally better.

External / Research-Linked

- External HTA agencies and affiliated initiatives contribute structured, policy-driven definitions of UMNs.
- IQWiG doesn't formally define UMNs but evaluates **added benefit** using mortality, morbidity, and QoL metrics. **Convenience or adherence** improvements are not valid unless tied to outcome gains.
- HTA skepticism of **surrogate endpoints** is strong; only validated ones are acceptable.
- The **NEED Project (Belgium)**, while not an HTA body, supports Belgian HTA with methodological input.
- It defines three levels of unmet need:
  - **Health needs** (clinical outcomes)
  - **Healthcare system needs** (service, side effects)
  - **Social needs** (employment, functioning)
- NEED promotes the concept of **"future unmet needs"**, based on projected system strain and disease burden.
- The project argues that **definitions limited to survival and morbidity** are inadequate — **QoL and social impact must be considered**.

- For conditions like **Crohn's disease**, NEED proposes multi-dimensional evidence of improvement (toilets in workplace), not just clinical response.

Note: The NEED Project is not an HTA agency but plays a key supporting role in shaping HTA frameworks and informing policy in Belgium.

Patient Insights / RWE

Internal

Patient-experienced professionals and RWE leads emphasize that UMNs must reflect the **real impact on people's lives**:

- Unmet need persists when symptoms are **incompletely controlled**, or treatments cause high emotional or social burden.
- Patients frame need in terms of **how a disease shapes their ability to live normally**, including emotional and social life.
- **Trained patient experts** should contribute to defining UMNs — not just as feedback, but as co-decision makers.
- Treatment burden (e.g., **side effects**, ongoing therapy fatigue) can itself constitute an unmet need.
- Some advocate for **longitudinal burden measurement** — not just peak severity, but persistence and relapse cycles.

1.2. Definition according to different expert groups

Clinical Stakeholders

Internal

- UMNs are defined as **gaps in efficacy, safety, or tolerability** of current therapies
- A treatment does not eliminate the unmet need if the **patient still experiences burden or symptoms**
- Definitions often relate to how far patients are from being "as healthy as possible" or able to function in daily life
- The **balance between therapeutic benefit and side effects** is central in identifying UMNs
- Disease subpopulations with poor or no treatment response are seen as key indicators of UMNs
- Some interviewees emphasize the need for **understanding the natural history** and individual patient profiles to define UMNs properly
- UMNs are **motivators for initiating drug development**, especially in early pipeline stages
- Expanded attention to **daily functioning and long-term treatment burden** gives broader meaning to "need"

External

- UMNs are identified when a disease or symptom is **not adequately treated by existing therapies**
- Even partial treatment efficacy may still represent a UMN if it **doesn't address the key patient outcomes**
- There is a preference for **binary criteria**: either treatments exist and work, or they don't
- When no treatment exists at all, the **definition of UMN becomes clear and uncontroversial**
- Focus is placed more on **clinical endpoints** (e.g., disease control, progression) than on broader QoL measures
- Emphasis on **regulatory simplicity**: unmet need must be demonstrable in a direct, clinical sense

Regulatory Affairs

Internal

- The **extent of the unmet need** influences whether drug development is justified
- UMNs are central for accessing **accelerated pathways and regulatory opportunities**
- Some see UMNs as potentially **subjective or divergent in interpretation**, depending on the stakeholder
- Definition is linked to **functionality loss, quality of life, and survival**, not just symptom management
- Practical consideration of **feasibility, investment, and regulatory gain** also shapes whether something is labelled a UMN
  Expanded

External

- UMNs must involve **life-threatening or seriously debilitating conditions**
- Must be assessed **case-by-case** with clinical judgment — not a one-size-fits-all definition
- When treatments are available, it must be shown that they don't **adequately improve survival, QoL, or function**

- Emphasis on **measurable outcomes**: burden reduction, survival extension, functional restoration
- Conditions with **uncertain benefit/risk balance** (e.g., due to lack of data) are also considered UMNs if impact is high
- Rare disease contexts may permit **flexibility in defining unmet need** due to data limitations

HTA / Market Access

Internal

- UMNs are assessed using dimensions like **efficacy, safety, and patient convenience**
- Existing treatments do not rule out UMNs when there is **residual burden** or unmet needs in subpopulations
- UMNs provide a **strategic entry point** — allowing room to operate commercially and clinically
- Definitions are shaped to **align with HTA expectations** (e.g., high burden, poor outcomes)
- Market viability depends on proving that current options are **insufficient from a societal or clinical value lens**
- Even if treatments exist, their **side effects or limitations** help define space for innovation
- Commercial framing also considers **payer sensitivity and burden rankings** across diseases
- Greater attention in transcripts to **data-driven framing for HTA value dossiers** (comparators, endpoints)

External

UMNs are validated only if there is proven added benefit over current therapies (e.g., survival, QoL)

- Treatment satisfaction or adherence alone is insufficient unless they translate into outcomes
- Frameworks avoid the term “medical need” — prefer “unmet health-related needs” to include non-medical gaps
- Unmet needs may include social, environmental, and psychological elements (e.g., toilet access, employment)
- Disease burden must be seen through a multi-layered lens: clinical, societal, future risk
- Concept of UMNs must be anchored in measurable impact, not assumptions of suffering

Patient Insights / RWE

Internal

- UMNs include **emotional, social, psychological, and functional dimensions**, not just clinical
- Burden must be interpreted through **lived experience**, not just clinical data
- Patients don’t separate disease from its **life context** (e.g., work, mobility, stigma)
- A disease may be "treated" but still represent a UMN if the **individual feels unwell or limited**
- Advocates for **patient-led prioritization** of what constitutes an unmet need
- Highlighted that **definitions vary by function** (e.g., advocacy, RWE, market access)
- More detailed illustrations in transcripts of **symptom-specific burdens** (e.g., sleep, mobility) not emphasized in summary
  Expanded
  Need for flexible, evolving definitions and regulatory clarity

Clinical Stakeholders

Internal

- **Definitions must be flexible, not prescriptive:** Fixed definitions may restrict curiosity and responsiveness to new observations, especially in rare diseases. Flexibility enables clinicians to adapt definitions to evolving contexts.
- **Regulatory clarity helps alignment but must allow for complexity:** Clear definitions help internal teams align with regulatory expectations, but if too narrow, they can suppress valuable nuance or localized insight.
- **Meaningful benefit and unmet need must be context-dependent:** Terms like “meaningful” or “unmet” vary by disease and stakeholder—more specificity and calibration are needed.
- **Burden perception differs between clinicians and patients:** Clinicians may overlook or undervalue symptoms that patients consider most impactful; this impacts how UMNs are identified.
- **Orphan designation should not be the only lens to define high UMNs:** There are serious UMNs in non-orphan diseases, and the assumption that orphan = high UMN is misleading.

- **More flexible criteria needed during early development stages:** Over-defining UMNs too early in development may reduce adaptability; definitions should evolve with ongoing research and emerging data.

External

- **UMN is strategically vague in pharma planning:** Lack of early clarity makes it hard to guide development; strategic justifications often come late in the process.
- **FDA's flexibility enables wide interpretation of UMNs:** FDA guidance is broad enough that most products can be positioned as addressing an UMN, whether or not it's truly high-need.
- **Clarity from regulators could help prioritize real need:** Looser definitions can lead to opportunism; more grounded criteria could help industry focus on truly urgent needs.

Regulatory Affairs

Internal

- Flexibility is higher for high UMNs, but this creates imbalance: Incentives and accelerated pathways exist for high UMNs, possibly at the expense of moderate or neglected areas.
- Definitions must be patient-driven but open to interpretation: Precision can hinder innovation; definitions must allow room for dialogue and disease-specific insight.
  External
- **Statistical and methodological support helps ground UMN claims:** Even if the need is clinically asserted, rigorous validation is needed to ensure the methodology supports it.
- **FDA leaves significant discretion to developers:** UMNs can be constructed around almost any case under current guidance, especially in early-phase filings.

HTA / Market Access

Internal

- **UMNs should be ranked by severity and population impact:** Stratification (e.g., low/medium/high) is essential for prioritization and access planning.
- **Tools like weighted scoring or indexing could support value communication:** HTA needs clearer quantification of how new treatments improve over current standards.
- **Real-world relevance outweighs narrow regulatory success:** Surrogate endpoints might get regulatory approval but fail to prove benefit in HTA reviews—focus must shift toward long-term outcomes. expanded
- **Disease evolution and societal values should influence definition updates:** As public health priorities change, so should the benchmarks used to define UMNs.

External

- **UMN frameworks must evolve with public health needs:** Changing conditions (e.g., pandemics, mental health trends) necessitate updated definitions and evaluative criteria.
- **R&D is biased toward oncology; underserved areas (e.g., mental health) need framework inclusion:** UMNs must reflect actual need, not just where the market currently focuses.
- **Definition differences between regulators and HTA bodies create inefficiencies:** When EMA and HTA bodies like IQWIG use different criteria, it delays or blocks access post-approval.

Patient Insights / RWE

Internal

- **UMNs evolve over time and must be reassessed:** Definitions must be dynamic to reflect emerging evidence, patient needs, and new treatments.

- **Patients and clinicians prioritize different symptoms:** For example, in Parkinson's, physicians focus on motor symptoms, while patients emphasize cognition—this misalignment matters.
- **Lack of continuity in teams weakens how UMNs are tracked internally:** As drug development teams change, there's risk of forgetting or miscommunicating the initial needs identified.
- **Definitions must include cases of treatment inaccessibility or intolerance:** Even if a drug exists, if patients cannot tolerate or access it, that's still an unmet need. expanded
- **Measurement trade-off: Generalizable tools vs. disease-specific sensitivity:** Broader tools risk missing real burdens; more granular tools may lack credibility across contexts. expanded

2. Influence on drug development

2.1. Impact of regulatory decisions on development and strategy
Clinical Stakeholders
Internal

- **Regulatory timelines and clarity shape project investment decisions:** If there's no predefined regulatory path, early-stage programs are often halted due to risk
- **Trial design must now address both regulatory and HTA demands:** clinical teams are adapting trial endpoints and duration to meet not only approval criteria but also reimbursement expectations. Expanded
- **New definitions of unmet need don't change current strategy:** UMN criteria are perceived as formalizations of existing assumptions; no major shift in development logic
- **Expedited programs are valuable to accelerate engagement:** Seen as helpful to fast-track dialogue with regulators and reduce development timelines
- **Desire for more flexible, less prescriptive regulatory environments:** There's some internal pushback against rigid expectations; teams would prefer more room for interpretation

External

- **Expedited programs are perceived as limited in real impact:** Despite designations like BTD, actual regulatory standards remain unchanged; these programs are seen more as symbolic
- **Skepticism toward regulatory leniency:** There's a sense that FDA still applies the same scrutiny regardless of expedited status, diminishing their strategic value
- Rigid regulatory expectations may constrain clinical innovation: Some external stakeholders feel agencies focus too much on predefined endpoints or processes, stifling more creative or adaptive approaches to development.

Regulatory Affairs Stakeholders
Internal

- **UMN helps frame borderline trial results for regulators:** When efficacy is mixed, unmet need can justify positive risk-benefit arguments
- **Programs addressing UMNs receive greater attention and resources:** Internal awareness that EMA and others may deprioritize assets outside high unmet need areas

External

- **UMN determination relies on clinical judgment and lack of alternatives:** MHRA emphasizes the role of physicians in identifying UMNs, and considers benefit-risk balance essential
- **Evidence quality must match UMN claims:** Claims of unmet need without robust data (e.g. single-arm trials) undermine credibility and slow regulatory progression

- **RWE is cautiously accepted under guidance frameworks:** MHRA offers frameworks for RWE, but insists on methodological robustness
- **Expedited labels don't guarantee easier regulatory treatment:** FDA still applies high standards despite expedited designations

HTA / Market Access Stakeholders

Internal

- **HTA assessments demand added value beyond regulatory success:** HTAs require demonstration of real-world benefit and quality-of-life impact; surrogate endpoints often fall short. [Expanded via transcript]
- **Comparator selection is strategic for HTA positioning:** Using weak comparators might ease regulatory approval but backfires during HTA evaluations, especially in DE and FR.
- **Internal misalignment on what constitutes success:** Clinical teams focus on regulatory approval, while access teams emphasize pricing and long-term viability.
- **Definition alignment between regulators and industry would streamline strategy:** A shared unmet need definition between pharma and regulators would enhance internal evaluations and BD alignment.
- **HTA input is increasingly influencing trial design:** Phase 3 planning is shaped by HTA demands; reimbursement strategy is built into design.

External

- **HTAs require different evidence than regulators:** While regulators prioritize benefit-risk, HTAs assess comparative value over existing treatments.
- **Robust, comparative trials are required:** HTAs expect head-to-head RCTs; indirect comparisons require strong justification.
- **Conditional approvals result from insufficient evidence:** HTAs may request additional data, conditionally approve, or reject added benefit.
- **Hard endpoints are non-negotiable:** Only outcomes like QoL, mortality, and morbidity are accepted; surrogates are rarely sufficient.

Patient Insights / RWE Stakeholders

- Clinical endpoints may not reflect patient experience: Anatomical improvements may not align with what patients value (e.g., less pain, more functionality). Expanded via transcript]
- Integration of RWE and PROs is growing, but uneven: There is growing interest in integrating patient-reported outcomes and real-world evidence, though planning is inconsistent. [Expanded via transcript]

2.2. Aligning challenges with regulatory expectations

Clinical

Internal

- Trial design must align early with regulatory expectations: Development programs are only pursued when there's a foreseeable regulatory path, especially for rare or early-phase conditions. [Expanded via transcript]
- Lack of validated tools limits ability to address certain UMNs: When no validated measurement exists, even high-priority needs become hard to progress into trials.
- Regulatory endpoints often don't reflect real-world patient priorities: Biomarkers required by regulators (e.g., urinary cortisol) may be less relevant to patient experience and clinical decision-making. [Expanded via transcript]
- Secondary endpoints are used to supplement regulatory inadequacies: When primary endpoints aren't convincing or meaningful, secondary measures are leveraged to demonstrate impact on UMNs.
- Scientific innovation blocked when no regulatory precedent exists: Promising biological concepts are deprioritized if there's no clear regulatory route to approval or

precedent for the condition.
[From transcript]

- Limited patient integration into early endpoint decisions: Although considered important, patient input is rarely embedded in early endpoint or trial design processes.
[From transcript]

External

- **Early trial phases rely on endpoints not recognized by regulators:** Phase I/II trials frequently use mechanistic or translational endpoints that diverge from what regulators eventually require.
- **Early cross-stakeholder planning (clinical, regulatory, commercial) is essential:** Trial design must balance multiple perspectives from the beginning to avoid misalignment later.
- **Rare disease designations support alignment and feasibility:** UMNs in rare diseases benefit from regulatory incentives (e.g., orphan status) that make development more feasible.

Regulatory Affairs

Internal

- **Cross-functional internal planning essential for regulatory viability:** Regulatory and research teams collaborate early to assess feasibility and clarify acceptable pathways.
[From transcript]
- **Rigid UMN definitions would be harmful—flexibility is crucial:** Definitions should leave space for severe or rare cases that may not be life-threatening but still urgent.
[From transcript]

External

- Robust methodologies are required despite UMN designation: High unmet need does not justify low-quality evidence; rigorous standards still apply.
- Trial design is less important than quality of evidence generated: It’s not the structure of the trial but the strength of the data that matters most to regulators.
- Early regulatory engagement (FDA, EMA) improves development success: Programs like ILAP or early advice mechanisms help shape trials around feasible, acceptable designs.
- Cross-region differences complicate global regulatory planning: EMA, FDA, and MHRA may vary in definitions, expectations, and pathways, complicating multi-regional planning.
[From transcript]

HTA / Market Access

Internal

- **HTA assessments demand added value beyond regulatory success:** Approval alone isn’t sufficient; HTA requires evidence of real-world benefit, cost-effectiveness, and patient-relevant outcomes.
[Expanded via transcript]
- **Comparator choice influences both HTA success and trial credibility:** Using weak or inappropriate comparators may satisfy regulators but undermine the case for added value in HTA reviews.
[From transcript]
- **Disconnection between functions on how to demonstrate UMNs:** Regulatory and access teams may agree on what constitutes an UMN but differ on what evidence is needed to demonstrate it.
- **Fast-track approvals create downstream HTA risks:** Design choices made for accelerated approval may compromise data robustness for HTA evaluations.
- **Endpoints may be outdated or unspecific for HTA use:** Common metrics (e.g., SF-36) may be inadequate for HTA needs in neuropsychiatric or rare conditions.
[From transcript]

External

- **HTA relies on data not designed for its purpose:** Companies typically submit regulatory data, which often lacks the necessary context or comparators for HTA assessment.
- **Indirect comparisons accepted but require high methodological rigor:** HTA bodies allow them, but they demand rigorous adjustment and can be more costly than a new RCT.
- **Pragmatic RCTs in registries (e.g. Nordic model) offer high-quality RWE:** Such designs are favored by HTA bodies when real-world evidence is needed for value demonstration.
- **HTA-regulatory planning should happen from development start:** Delaying HTA strategy until post-approval increases misalignment risk.
- Approval ≠ reimbursement without strong economic and clinical value case: HTA rejection still occurs if the product doesn't offer meaningful improvements or is priced too high.

Patient Insights / RWE

Internal

- **Fragmented HTA-regulatory alignment harms patient access across countries:** Even within Europe, different definitions and evidence standards block equal access to therapies.
- **RWE is gaining traction but must be rigorous and relevant:** Only methodologically robust RWE (e.g., from registries or pragmatic trials) is useful in decision-making.

[Expanded via transcript]

- **Patient voice inconsistently embedded in development strategy:** Despite progress, patients are often consulted too late to shape trial goals or outcomes. [From transcript]

2.3. Balancing scientific feasibility with UMNs (choosing or designing scientific projects that are both doable and meaningful)

Clinical Development

Internal

- **Scientific feasibility acts as a decision filter**: Even in areas of clear unmet medical need, internal clinical teams often avoid advancing compounds if demonstrating benefit appears unfeasible — due to complexity, cost, or regulatory uncertainty.
- **Biological translatability limits feasibility**: Development begins from a biology-first model, but even strong mechanistic understanding may not translate into effective treatments, particularly in neurological or rare diseases.
- **Feasibility challenges intensify in rare diseases**: While smaller trials are acceptable, they come with limited data, unclear endpoints, and higher uncertainty. This makes scientific and regulatory feasibility much harder to establish.
- **Endpoints often lack relevance or validity**: Trials must often rely on surrogate or generic measures (like 6-minute walk test) that do not reflect patient-prioritized symptoms. Developing better endpoints is expensive and risky.
- **Patient symptom heterogeneity complicates feasibility**: Patients with the same diagnosis may suffer from entirely different symptom burdens. This makes endpoint standardization and trial generalization difficult.

External

- **Small patient groups reduce evidence translatability**: In early development, especially for rare diseases, feasibility is hampered by weak or uncertain signals that are insufficient to justify full development.
- **Feasibility alone doesn't justify an UMN in saturated fields**: In well-treated areas like depression or schizophrenia, justifying a program as an unmet need requires a very specific patient subgroup or treatment resistance argument.

Regulatory Affairs

Internal

- **Feasibility tied to likelihood of regulatory success**: Regulatory strategy must be defined early to assess if a program will realistically meet evidentiary thresholds. Otherwise, a scientifically feasible idea may never translate into a file.

- **Regulatory standards vary regionally, impacting feasibility**: EMA is often more rigid than FDA, making feasibility context-dependent based on geography and incentive schemes.
- **Addressing subpopulations raises feasibility barriers**: Regulators often push for broader treatment populations than the drug is designed for, which can make development unfeasible.

External

- **Regulators value robustness of evidence over design type**: Flexibility exists in acceptable trial designs — including single-arm and real-world studies — but only when evidence is unambiguous and strong
- **Early engagement improves feasibility**: Involving statisticians and regulators in early-stage planning ensures scientific approaches are aligned with regulatory expectations.
- **One strong pivotal trial may suffice — if backed by Phase 2**: This can reduce time and cost, but increases pressure on early-phase feasibility.
- Scientific feasibility ≠ HTA feasibility: Regulatory acceptance doesn't guarantee that value can be demonstrated for reimbursement. This limits the practical feasibility of some programs.

HTA / Market Access

Internal

- **Feasibility is assessed early with payer perspectives in mind**: Projects are evaluated early on whether they can succeed not only clinically and regulatorily, but also economically.
- **Innovative endpoints are rarely pursued**: Despite their relevance to UMNs, companies avoid proposing new endpoints due to their cost, risk, and low chance of success with payers or regulators.
- Regulatory approval ≠ reimbursement guarantee: Payers may reject drugs based on design flaws (e.g., comparator issues), even when regulators have fast-tracked or approved them.
- **Real-world feasibility is often missing from early planning**: Trials that succeed under idealized trial conditions often fail to translate to real-world patient behavior, adherence, or benefit.

External

- **UMNs must be system-level and evidence-driven**: Companies must prove that the need exists not just theoretically but in real health system data, or risk investing in the wrong priorities.
- **Scientific feasibility must match population burden**: Feasible development plans that aren't aimed at the most burdensome conditions or subgroups will struggle to gain support.

Patient Insights / RWE

Internal Stakeholders

Key Ideas:

- **Feasibility depends on aligning endpoints with lived experience**: If outcomes measured in trials do not reflect patient priorities, drugs may be seen as ineffective, even if scientifically sound.
- **Patient engagement improves feasibility and endpoint selection**: Involving patients early helps identify which symptoms truly matter, especially in diseases with variable presentations.
- **RWE can support more realistic identification of UMNs**: Combining population-level data with patient input helps define feasible, justified, and real unmet needs.

2.4. HTA considerations

CLINICAL STAKEHOLDERS

Internal

- **HTA requires designing trials beyond regulatory goals:** Clinical teams increasingly recognize that regulatory trials alone are not sufficient for achieving

reimbursement. Trials must also incorporate endpoints that reflect QoL, daily functioning, or cost implications to meet HTA expectations.

- **Rare diseases highlight complexity in HTA alignment:** Heterogeneous symptoms and lack of validated endpoints in rare diseases make it difficult to demonstrate value in a standardized way. HTA strategies must adapt to the lived experiences of patients with diverse manifestations.
- **Clinical differentiation is essential, not just regulatory approval:** HTA decisions demand more than efficacy—products must be meaningfully different in real-world performance and address needs unmet by cheaper alternatives.
- **Early internal collaboration with commercial functions is lacking:** Although evidence requirements for HTA are known, alignment between clinical and commercial planning is still not systematic, delaying value-based trial designs.

External

- **HTA definitions of value are grounded in cost-effectiveness logic:** Even if a drug demonstrates scientific validity, payers assess whether it reduces burden in measurable, economic terms, like reduced hospital stays or improved workforce participation.
- **HTA and regulatory bodies apply different standards for UMNs:** While FDA designations may focus on severity or rarity, HTA agencies demand comparative value against available options, often disqualifying drugs seen as marginal improvements.

REGULATORY AFFAIRS

Internal

- **Regulatory approval is no guarantee of patient access:** Even when benefit–risk is favorable, HTA bodies may still deny reimbursement based on economic or societal thresholds, creating pressure to align earlier.
- **Cross-functional integration is critical for HTA readiness:** Regulatory teams are advocating for earlier engagement with commercial and market access colleagues to plan submissions that anticipate HTA demands.

External

- **Joint scientific advice helps align evidence plans across stakeholders:** More HTA bodies now collaborate with regulators to offer early guidance, helping companies shape trials that serve dual requirements.
- **Patient engagement improves evidence relevance for HTA:** Involving patients helps highlight which outcomes matter most for quality of life, ensuring HTA decisions reflect real burdens—not just clinical endpoints.
- **HTA logic diverges from regulatory flexibility:** Even accelerated approvals (e.g., PRIME, Fast Track) can lead to HTA rejection if data lacks robustness or comparative relevance.

HTA / MARKET ACCESS

Internal

- **HTA assessments demand added value beyond regulatory success:** Approval based on surrogate or narrow endpoints is insufficient—HTAs require proof of real-world benefit, cost-offsets, and quality-of-life improvements.
- **Comparator selection is strategic for HTA positioning:** Choosing weak or outdated comparators may ease regulatory approval but weakens value arguments during HTA assessment, particularly in Germany or France.
- **Early HTA alignment is becoming standard practice:** Access teams are increasingly embedded in clinical planning to ensure endpoints and comparators meet reimbursement thresholds from the outset.
- **Evidence must be framed persuasively for HTA 'negotiation':** Unlike regulators, HTA bodies respond to narratives. Value dossiers need to integrate physician interviews, burden data, and positioning strategies.
- **Internal disconnection hampers HTA success:** When clinical, regulatory, and market access teams operate in silos, HTA evidence can lack cohesion, reducing pricing and access opportunities.

External

- **HTA bodies require measurable clinical outcomes, not process improvements:** Unlike regulators, HTAs won't credit workflow benefits unless they translate into tangible reductions in morbidity, mortality, or QoL deficits.
- **Surrogate endpoints undermine HTA evaluations:** Drugs approved based on non-validated surrogates often fail to show added benefit under HTA scrutiny, especially in Germany or Belgium.
- **Economic and patient burden data is increasingly expected:** Belgium and other EU HTAs are formalizing requirements for companies to provide burden and UMN evidence before pricing discussions begin.
- **Engaging in early HTA dialogue is becoming increasingly important: without it, companies may face challenges if trial designs fail to align with unmet need criteria.** Expectations around demonstrating value are shifting toward sponsors.

PATIENT INSIGHTS / RWE

Internal

- **Access fragmentation persists despite EMA approval:** Even after regulatory success, patients may face delays or denials due to national HTA or pricing decisions.
- **Uneven HTA standards across countries raise equity concerns:** When similar products receive divergent HTA outcomes across the EU, it raises ethical concerns about fairness in patient access.
- **RWE generation is expensive and under-recognized:** The effort to collect patient-relevant, real-world data for HTA review is substantial—and companies bear most of that burden.
- **Scientific patient input is key to endpoint design:** Capturing what matters to patients, in structured and validated ways, is essential to shaping HTA-relevant evidence strategies.
- **Generic QoL tools often miss disease-specific burdens:** Standard instruments like EQ-5D offer cross-disease comparability, but may fail to capture what truly matters to specific patient groups.

2.5. UMNs considerations in drug development

Clinical

Internal

- **UMN identification must start early, ideally in research stage:** Stakeholders consistently emphasize that programs are only initiated if a clear unmet need is visible from the outset, often as a formal requirement to enter the pipeline.
- **Scientific feasibility and biological rationale drive investment before formal UMN framing:** Scientific novelty or mechanistic plausibility is prioritized; unmet need is considered valid only if it aligns with clear biological targets and tractable clinical translation.
- **Trial design must balance regulatory and payer demands from the start:** Protocols are increasingly designed with HTA, regulatory, and patient outcomes in mind—not just to demonstrate efficacy but to align with future access and perception of value.
- **Severity, patient burden, and lack of durable response are core to defining an UMN:** Stakeholders stress that it's not enough to show efficacy—real unmet need involves clear, sustained improvement in function or reduction in suffering.
- **Perceived UMNs must be clinically demonstrable within accepted endpoints:** Many biological opportunities are not pursued if endpoints are lacking or regulatory recognition is uncertain, regardless of scientific merit.
- **Patient input is inconsistently integrated but increasingly valued:** Efforts are underway to gather broader patient insights, but systematic integration into trial design or disease prioritization remains limited.

External

- **UMNs are used strategically to qualify for expedited pathways like Breakthrough or PRIME:** Early regulatory engagement often revolves around showing that a program addresses an unmet need to unlock special designations.
- **Demonstrating UMN is often more difficult when there are existing treatments with partial effectiveness:** In areas like psychiatry or pain, proving that existing treatments are insufficient remains a challenge—UMN arguments often hinge on tolerability, subpopulations, or adherence.

- **Misalignment between regulatory approval and HTA access often stems from vague or unsubstantiated UMN claims:** Even when regulatory approval is granted, reimbursement can be denied if HTAs dispute the unmet need justification.
- **Scientific rationale is not enough—agencies and payers require structured evidence of benefit in real populations:** Proving that an intervention addresses a meaningful UMN requires more than novelty; it must show measurable, practical patient outcomes.

Regulatory

Internal

- **Companies struggle to align internal priorities with regulatory expectations on UMNs:** While internal teams might pursue biologically attractive programs, they don't always match the EMA/FDA's definition of unmet need, which can block approval pathways.
- **UMN framing gains importance when trial results are borderline:** Regulatory flexibility (e.g., conditional approval) often hinges on the severity of the unmet need—especially when primary endpoints are not fully met.
- **There is no standard formula for defining UMNs internally—decisions remain subjective:** Clear definitions are absent even at internal portfolio review level; each case is evaluated on an ad hoc basis, often based on cross-functional judgment.
- **Regulatory and business incentives around UMNs are not always aligned:** Portfolio decisions sometimes favor avoiding markets where regulatory hurdles tied to UMN definitions reduce commercial potential.

External

- **Regulators use UMNs to justify flexibility in data requirements and approval conditions:** High unmet needs can justify conditional approvals, smaller trials, or post-market evidence requirements, but only when solid rationale is presented.
- **Early engagement programs like ILAP are used to explore and validate UMN framing:** Tools like ILAP, PRIME, or Breakthrough allow early feedback, but require robust data to back claims.
- **UMNs are defined differently by regulators and HTAs—bridging this gap is crucial:** Approval without access remains a risk unless both parties agree on the need, which often doesn't happen.
- **Statistical considerations can complicate regulatory recognition of UMNs in rare or heterogeneous populations:** Designing studies that meet regulatory standards in UMN areas (e.g., neurodegeneration) is especially challenging.

HTA / Market Access

Internal

- **HTA teams assess UMNs not just on clinical need but also on comparative advantage and QoL gains:** Programs must show they outperform alternatives in real-world dimensions that matter to payers and patients—not just meet regulatory efficacy bars.
- **UMNs are increasingly integrated in early-stage access strategy decisions (Ph1/2):** Market access specialists are consulted as early as phase 1 to ensure unmet needs are valid, measurable, and aligned with future submissions.
- **Misalignment between internal enthusiasm and payer expectations can derail otherwise promising assets:** Therapies with scientific merit may be deprioritized if unmet need cannot be convincingly demonstrated to HTA bodies.
- **Incentive-driven development (e.g., rare diseases) risks overlooking true public health needs:** Internal stakeholders are aware that pursuing rare/orphan routes for UMN claims may lead to crowded pipelines without addressing broader population needs.

External

- **HTA bodies require measurable clinical outcomes, not process improvements:** Unlike regulators, HTAs won't credit workflow benefits unless they translate into tangible reductions in morbidity, mortality, or QoL deficits.
- **Definitions of UMNs must be evidence-based and consistently applied—HTAs are pushing for formal frameworks:** HTA bodies demand early, data-supported UMN claims to be made part of pricing and reimbursement discussions.

- **HTA and regulatory standards are not harmonized, leading to systematic barriers to access post-approval:**
 Discrepancies in what qualifies as an UMN often delay or prevent market access.
- **Future incentive systems must distinguish between commercial rarity and societal need:**
 Stakeholders warn that current incentives encourage companies to target "UMNs" that fit regulatory definitions but lack meaningful public health impact.

Patient Insights / RWE

Internal

- **Real-world data and patient insights are underused in early UMN identification—but this is changing:**
 Efforts are growing to incorporate qualitative and observational data into early strategy and trial design, especially in neuropsychiatric diseases.
- **Patients often prioritize outcomes (e.g., brain fog, agitation, function) that lack validated endpoints—limiting UMN recognition:**
 Patients' unmet needs frequently focus on symptoms overlooked in traditional trials, hindering demonstration of benefit.
- **Collaborating with broader patient communities, not just "super patients," helps reveal neglected UMNs:**
 Internal teams acknowledge a bias toward highly informed, engaged patients and are working to reach more representative populations.
- **Quality of life impact, caregiver burden, and functional independence are central to real UMNs:**
 Beyond clinical measures, real unmet needs often lie in preserving life roles and daily functioning.

3. Stakeholder Insights

3.1. Role of patients, clinicians, and payers in defining and prioritizing UMNs

1.1.1. Incorporating patient and clinician feedback into development and regulatory strategies

1.1.2. Market access as a stakeholder

Clinical Development Stakeholders

Internal

- Clinician input ensures trial feasibility and endpoint development
 Clinicians are essential to assess whether trials are implementable and to co-develop meaningful biomarkers.
- Broadening patient input is still maturing
 Input from patients is increasingly integrated but considered more difficult to systematize than clinician input.
- Caregiver and family perspectives are valuable, especially in rare indications
 Family input often uncovers overlooked symptoms and burdens that shape development strategy.
- Engagement of patients through advisory boards and interviews throughout development
 Used pre-trial (to test endpoints), during trials, and post-trial to refine interpretation.
- UMNs must align with biological feasibility and clear clinical translation
 From a research view, conditions are prioritized if mechanisms are actionable and trial designs feasible.
- Real-world relevance requires early patient-centered thinking
 There's increasing awareness that symptom burden should guide development choices, not just biology.

External

- **Patient input exposes hidden gaps not seen in clinical protocols**
 Patient experiences often highlight issues not reflected in diagnostic criteria or standard.
- **Clinicians and patients often diverge on what matters most**
 Historic mismatch, but increasing alignment due to patient-focused regulatory frameworks (especially in FDA).
- **Engagement with families is standard practice in rare/psychiatric conditions**
 Especially in early-phase regulatory work, family input added insight on what truly matters.

Regulatory Affairs Stakeholders

Internal

- **Patient engagement happens through advocacy groups or trial participation**
  Information gathered from observational trials and structured interviews feeds into regulatory positioning.
- **Clinical advisors and KOLs help refine development from a regulatory view**
  Used to shape alignment of trials with UMN narratives.
- **Patients may bring up symptoms overlooked by clinicians but hard to align to biological markers**
  Their inputs may not always fit regulatory endpoints but can still enrich understanding.

External
- **Patient engagement improves trial retention and relevance of outcomes**
  Early engagement leads to better safety reporting and trial continuity.
- **Involving patients helps define regulatory-relevant outcomes**
  Outcomes important to patients (e.g. function, pain) are being increasingly considered in benefit-risk.
- **Regulatory agencies institutionalize patient involvement**
  Through formal roles and meetings, especially in UK and US regulatory systems.

HTA / Market Access Stakeholders
Internal
- **HTA bodies scrutinize proof of value, not just regulatory approval**
  Approvals based on biomarkers or expedited designations often fail to secure reimbursement.
- **Patient input alone has limited impact unless combined with media or political pressure**
  HTAs rarely change decisions unless broader societal attention is applied.
- **HTA decisions focus on societal burden, cost-effectiveness, and real outcomes**
  Not just on whether a treatment technically qualifies as addressing an unmet need.
- **Patients and clinicians must help quantify value in HTA terms (functionality, quality of life)**
  Especially in psychiatric or rare diseases where traditional metrics fall short.
- **Payers should be involved earlier to align clinical development with access hurdles**
  Delayed payer involvement creates misaligned endpoints and blocks reimbursement.

External
- **Patient input is formally captured in HTA via questionnaires and hearings, but no voting rights**
  In Germany, patients contribute views, but influence is mostly indirect.
- **Structured methodologies exist for UMN assessment using multi-stakeholder input**
  Includes clinicians and patients through Delphi processes and expert tools.
- **Incorporating patient views into HTA frameworks is evolving (e.g., pediatric focus, sexual health impacts)**
  Frameworks are adapting to include diverse perspectives.

Patient Insights / RWE Stakeholders
Internal
- **Patient input brings essential lived experience, shaping what qualifies as a 'real' unmet need**
  Patients provide insight into burdens not reflected in traditional data.
- **Every interaction with patients reveals new needs, making UMNs a dynamic, evolving concept**
  Needs shift over time and between individuals.
- **Earlier engagement with patients now becoming more systematic**
  It used to be a challenge, but now companies engage patients from early phases (e.g., Phase 1). [Expanded via transcript]
- **Expert patients (not average ones) are especially useful for early R&D strategy**
  These individuals help define what should be prioritized and why.

3.2. Use of RWE to justify UMNs and align with medical needs
Clinical
Internal
- **RWE integration remains limited in early development phases, though its potential is recognized.**

While some departments are co-located with RWE experts to improve future integration, real-world data is currently underutilized in early submission packages.

- **Natural history studies and registries can strengthen unmet need justifications, especially in rare diseases.** Using real-world data (e.g., patient registries or historical controls) can justify medical need or serve as safety/placebo arms in rare indications.
- **RCTs dominate regulatory decisions; RWE remains complementary at best.** RCTs are still the standard for regulators and payers. RWE rarely influences pivotal decisions, but may help explain or contextualize findings.
- **Subjectivity in patient experiences complicates RWE interpretation.** Patient advocacy groups can overrepresent certain voices, making it difficult to generalize findings to broader populations.
- **RWE can highlight stigmatized or underreported issues.** E.g., impulse control disorders in Parkinson's revealed by Swedish registries were poorly addressed in trials due to stigma.
- **Patient preferences can shape evidence strategies.** Preference data (e.g., patients refusing older treatments due to side effects) could retroactively justify the value of newer drugs.
- **Not all biologically interesting concepts translate to unmet need.** Some mechanisms (e.g., motor symptoms in Parkinson's) are biologically novel but may not address significant gaps in treatment.

External

- **RWE used to formalize unmet need via registries, chart reviews, or payer data.** Companies may proactively document unmet needs using formalized RWE (prospective or retrospective).
- **RWE effective when formalized and prospective.** Regulators give more weight to prospective, structured real-world studies.
- **Post-marketing utility of RWE dominates current practice.** Pre-approval use is limited; RWE typically supports label expansions or post-approval modifications.
- **Using RWE to justify development of follow-up treatments.** Approved drugs can anchor RWE collection that supports subsequent product development.

Regulatory Affairs

Internal

External

- **RWE and PROs play a supportive but non-decisive role, especially in rare diseases.** While encouraged, RWE is not accepted as primary evidence; MHRA sees it as context-specific support.
- **RWE use requires robust, relevant, high-quality data.** Regulators stress methodological flexibility, but data quality is essential.
- **Statisticians can support RWE inclusion by ensuring methodological rigor.** No fixed approach is required, but analyses must be transparent and defensible.
- **Regulators act independently—acceptance of RWE in one agency doesn't imply alignment across others.**

5. HTA / Market Access

Internal

- **HTA relevance of RWE varies across markets, especially valuable in rare diseases.** Some HTA bodies give more consideration to RWE when RCT data is limited.
- **RWE improves trial generalizability and can support local relevance in payer evaluations.** It helps contextualize trial data to national populations.
- **Post-launch RWE is more impactful for HCP persuasion than for price setting.** HTA pricing is primarily influenced by pre-launch trial data.
- **Lack of clear HTA guidance limits RWE's use.** A formal weighting (e.g., "25% of value assessment") could increase consistency.
- **Internal push for RWE adoption exists but needs structure.** Teams recognize RWE's importance and try to integrate it more formally.

External

- **RWE must be rooted in local healthcare contexts to be useful.** Data from routine practice—not generic "RWE"—is preferred.
- **Observational RWE is weak for efficacy but strong for descriptive insights.** Useful for treatment patterns and population characteristics, but not causal inferences.
- **Patient-reported outcomes (PROs) are vital for HTA assessments.** Especially in orphan and psychiatric conditions.
- **Use of RWE is increasing but still seen as supplementary.** Growing recognition, but not yet primary data source.

7. Patient Insights / RWE

Internal

- **Small sample sizes in rare disease RWE reduce perceived validity.** Real-world studies in rare diseases are often questioned due to low statistical power.
- **Quantified patient voice should supplement general RWE.** RWE should combine easily accessible data (e.g., claims) with scientifically validated patient perspectives.
- **Discrete choice experiments help capture patient preferences.** Useful in HTA and health economics to express trade-offs between longevity and quality.
- **Collaborative, pre-competitive spaces can improve RWE quality and trust.** Multi-stakeholder frameworks are needed for better concept development.
- **RWE is underused but essential in capturing under-acknowledged needs.** Stigma and side effects are often invisible in trial data, but can drive patient behavior.
- **Patient preferences can influence regulatory/payer strategy if captured early.** Studies showing patients prefer no treatment over existing options help reposition newer alternatives.

3.3. Challenges in incorporating stakeholder insights

Clinical Development

Internal

- **Not all clinicians provide meaningful patient insight input:** High-profile clinicians may lack the time or orientation to deeply understand patient realities, whereas more observant or engaged physicians can offer invaluable insights
- **Trial design often favors measurable endpoints over meaningful ones:** Standard endpoints may fail to reflect the true burden or what matters to patients (e.g., migraine pain vs. frequency). This misalignment complicates translating needs into approvable endpoints
- **Patient feedback varies widely; "super patients" dominate discussions but don't represent the average patient:** Advisory boards may over-rely on articulate, highly informed patients. One-to-one engagement and broader patient recruitment strategies are needed to diversify inputs.
- **Integrating insights from clinicians and patients into trials remains difficult despite growing intent:** Though input is routinely gathered, there are gaps in translating it effectively into trial designs, especially when there's a lack of validated tools or endpoints in rare diseases.
- **Disease complexity and individual patient variability complicate endpoint selection:** Some symptoms (e.g., sleep problems, functional impairment) may be relevant only to subsets of patients, requiring more personalized or modular outcome measures.
- **Bias and data quality issues in current input methods:** Surveys, advocacy group data, and digital platforms introduce bias. Social media may distort what's "real," and advocacy input often excludes broader patient groups.

Internal group members not contributing:

External

- **FDA increasingly institutionalizes patient voice, but constraints remain:** Regulatory frameworks are beginning to mandate structured incorporation of patient insights (e.g., sections in NDAs), but there's still uncertainty about authentic representation.
- **Some advocacy groups may not reflect diverse patient experiences:** Sponsorship or over-structuring of advocacy efforts raises concerns about whether the feedback is fully representative.

External group members not contributing:

Regulatory Affairs

Internal

- **Lack of consensus on who defines the UMN bar:** Different authorities and stakeholders interpret unmet need differently, making it hard to apply consistent standards across jurisdictions.
- **Fragmentation between HTA and regulatory systems impairs insight integration:** Even with centralized approval, reimbursement decisions are made independently and may disregard regulatory reasoning.
- **Joint EMA–HTA advice mechanisms exist but are underutilized:** Opportunities for early alignment exist (e.g., parallel advice), but are still not routinely leveraged across teams or organizations

External

- **Regulatory openness to patient voice has increased (e.g., at FDA):** There's now formal space in regulatory reviews for patient experience data, but aligning it with evidence standards remains challenging.

HTA / Market Access

Internal

- **Patient input is still undervalued unless paired with political/media pressure:** Insights from patients are often treated as tokenistic unless public advocacy shifts the conversation politically.
- **Compliance rules hinder patient collaboration:** Strict rules around industry–patient interaction prevent meaningful involvement during access planning.
- **Mismatch between trial endpoints and real-world value frustrates HTA success:** Approval may be based on endpoints with weak real-world impact (e.g., biomarkers), while HTAs want QoL, symptom relief, or economic value demonstrated.
- **Early HTA engagement and outcome validation is key to derisking asset development:** Effective internal alignment and external consultations must occur early (e.g., pre-Phase 1), to align endpoints with payer expectations.
- **Comparator selection in trials critically affects HTA reception:** Using weak comparators may help with regulatory success but undermines value demonstration during HTA review.

External

- **KOLs excluded from formal HTA input to avoid bias:** HTA frameworks (e.g., IQWiG) restrict expert contributions due to COI concerns, potentially limiting clinically nuanced feedback.
- **Recruitment and insight gathering vary drastically by disease:** Populations like those with psychotic disorders pose significant recruitment challenges for patient insight collection.

Patient Insights / RWE

Internal

- **RWE integration requires pre-competitive, multi-stakeholder alignment:** Consensus frameworks developed before product-specific discussions can streamline HTA and regulatory alignment.
- **Emotional burden, silence, and systemic barriers limit patient input quality:** Many unmet needs remain unvoiced due to emotional toll, lack of medical framing, or limited alternatives.
- **Need for better tools to gather insights from unrepresented patients:** Patients outside organized advocacy structures are hard to reach and tend to be excluded from the insight pipeline.
- **In rare diseases, small sample sizes reduce RWE validity:** Real-world studies are often discounted due to insufficient numbers, especially in neurodegenerative conditions.

4. Regulatory alignment

4.1. Impact of regulatory definitions on portfolio prioritization

Clinical

Internal

- Regulatory categories (e.g., UMN/HUMN) are seen as influencing value positioning more than trial design.

- Concern over exclusion of chronic burdensome conditions due to narrow UMN definitions.
- Clinical teams often work around regulatory constraints by tailoring endpoints to patient-specific symptoms, especially in rare diseases.
- Regulatory recognition (e.g., ODD, PRIME) may retroactively validate clinical direction, but initial prioritization relies on other factors.
- Regulatory uncertainty can delay or redirect prioritization in rare disease programs.

External

- Unclear UMN definitions create misalignment across regions.
- Clinical developers use regulatory incentives (e.g., fast-track) tactically but remain skeptical of their consistency.

Regulatory Affairs

Internal

- Regulatory definitions rarely drive portfolio decisions directly—business factors override.
- Misalignment between EMA and business priorities creates tension—e.g., avoiding pediatric obligations can kill EU launches.
- Regulatory decisions indirectly shape risk-benefit assessments and market expectations.
- Subpopulation focus is often disincentivized by regulators who expect broad impact, making prioritization riskier.
- Regional divergence in definitions (e.g., EU vs US) complicates global strategy.

External

- Statisticians play a proactive role early in regulatory engagement.
- Regulatory categorization should better reflect patient impact and practical feasibility.

HTA / Market Access

Internal

- Regulatory designations (e.g., UMN) don't guarantee HTA acceptance—real-world outcomes matter more.
- Narrow regulatory definitions risk excluding chronic and prevalent conditions from incentives.
- Early collaboration across departments helps align regulatory and value strategy.
- Fragmentation of definitions and incentives across countries (esp. in EU) challenges development planning.
- Incentives shape prioritization, but not always enough to drive development if market returns are uncertain.

External

- HTA definitions of unmet need require measurable health outcome improvements—not just process improvements.
- Incentives tied to orphan designation (e.g., added benefit assumption) distort HTA assessment.
- Efforts to align HTA and regulatory definitions (e.g., in Belgium) exist but face practical hurdles.

Patient Insights / RWE

Internal

- Regulatory and HTA alignment is ideal but unlikely due to fragmented systems.
- Lack of harmonized definitions across regions leads to patient access inequality.
- Early regulatory engagement (e.g., ODD) is now better integrated in RWE teams' planning.
- Practical RWE and patient data must inform regulatory claims to strengthen the case for UMNs.

4.2. Differences in requirements (EMA/FDA/MHRA - HTA). Divergences/similarities.

Clinical

Internal

- **Divergences in regulatory expectations:** FDA tends to be more flexible in accepting biomarkers and surrogate endpoints, especially in rare diseases, while EMA requires more structured, long-term, and comparative evidence.
- **EMA expectations include economic and comparative logic, anticipating HTA concerns more than FDA:** EMA demands evidence not just of safety/efficacy but also of superiority over existing treatments, reflecting early integration of economic reasoning.
- **Rare diseases allow more creative trial designs (especially in US):** FDA encourages creativity in evidence generation for rare indications; Europe is more cautious.
- **Early regulatory consultation is necessary to anticipate alignment gaps:** Internal teams systematically engage early with regulators (FDA/EMA) to flag feasibility or red flag concerns.

External

- **Labeling flexibility varies across regions:** Europe has stricter rules on what can be included in labels; US labels allow more flexibility in narrative.
- **Regulatory flexibility shapes development strategies:** FDA allows earlier engagements and faster timelines (e.g., rolling submissions), creating incentive to prioritize US development.
- **Strategic divergence between regulatory and HTA value demonstration:** HTA needs go beyond clean data to patient-relevant outcomes, while regulators still prioritize technical efficacy/safety.

Regulatory Affairs

Internal

- **EMA requires higher evidence standard (comparators, 3-arm studies):** EMA generally requires head-to-head comparisons or active comparator trials, unlike FDA where such designs are often optional.
- **FDA allows more leeway in evidence and may accept post-marketing commitments:** FDA may defer evidence generation (e.g., confirmatory trials), offering more flexibility in approval pathway.
- **EMA and FDA have diverging definitions of what qualifies as “high unmet need”:** Different incentive triggers and designation rules (e.g., breakthrough vs. orphan) across agencies affect strategic planning.

External

- **MHRA and EMA are mostly aligned, but act independently:** Approval by EMA does not automatically translate to UK access; eligibility criteria for expedited pathways differ.
- **Statisticians assess evidence robustness—not UMN qualification:** At MHRA, unmet need status is determined clinically; evidence quality remains essential regardless of designation.
- **Rarity or UMN does not lower the evidentiary bar:** Even in rare diseases, claims of UMN do not lead to relaxed standards unless clearly justified.
- **FDA tends to accommodate early-phase flexibility:** Expedited programs in the US (e.g., breakthrough therapy) hinge on early evidence, even if full datasets aren’t yet available.

HTA / Market Access

Internal

- **HTA assessments demand added value beyond regulatory success:** Approval based on surrogate or narrow endpoints is insufficient—HTAs require proof of real-world benefit, cost-offsets, and quality-of-life improvements.
- **Comparator selection is strategic for HTA positioning:** Choosing weak or outdated comparators may ease regulatory approval but weakens value arguments during HTA assessment, particularly in Germany or France.
- **Lack of alignment creates strategic tensions:** A drug may be approvable but not reimbursable; early HTA consideration is critical.
- **Joint HTA-regulator initiatives exist but fall short in impact:** EMA-HTA early advice is available but underused; country-level autonomy persists in pricing/access.

External

- **HTA requires comparative and measurable benefit over standard of care:** HTAs demand outcomes that show added value beyond placebo or broad “patient care” claims accepted by regulators.
- **Orphan designation misalignments persist:** Orphan status may secure regulatory incentives but doesn’t guarantee HTA acceptance unless health outcomes improve.
- **HTA bodies operate independently despite JCA initiatives:** Even with joint clinical assessments, final reimbursement decisions remain national.
- **Lack of standardized patient-centric data weakens HTA submissions:** Despite growing awareness, companies often fail to plan for PRO and QoL data early enough.

Internal

- **Patient input is crucial but under-integrated into regulatory and HTA alignment:** Agencies increasingly value patient-relevant outcomes, yet these are often underused in early trial design.
- **Real-world evidence (RWE) is variably accepted:** FDA shows more openness than EMA/HTA to use RWE, especially in rare diseases; however, evidentiary standards must still be met. Expanded
- **Patient-reported outcomes are valuable but not consistently prioritized:** PROs are acknowledged as important, yet still seen as "supportive" rather than pivotal by some agencies. Expanded

4.3. Balancing regulatory clarity with flexibility to meet evolving medical needs

Clinical

Internal

- **Tension between regulatory clarity and real-world variability in rare diseases:** A rigid definition of UMNs risks excluding valid but atypical patient needs, especially in rare or heterogeneous diseases. Flexibility is crucial to allow for case-specific nuances
- **Patient relevance is hard to quantify but essential for alignment:** Clinical endpoints often fail to reflect what actually matters to patients (e.g., fatigue, daily activities), and regulatory clarity shouldn't ignore these lived experiences.
- **Regulatory granularity must be balanced with innovation:** Too much specificity in guidelines can undermine novel approaches and early-stage decisions. Clinical teams prefer adaptable standards that evolve with science. **Misalignment within companies on UMN implications:** Different internal teams prioritize regulatory alignment vs. real-world impact, which creates ambiguity in early-phase planning.

External

- **UMNs require early strategic framing but current definitions are vague:** The ambiguity around what counts as an UMN complicates early-stage clinical strategy and endpoint selection. A shared understanding would aid forward planning.
- **FDA's guidance lacks clarity on demonstrating alignment with UMNs post-designation:** Receiving a designation (e.g., BTD) does not ensure regulators will later agree the product addressed an UMN—more guidance is needed post-designation.
- **Flexibility must be scientifically justified:** Relaxed evidentiary standards should not be assumed; regulators demand robust data regardless of "UMN" status.

Regulatory Affairs

Internal

- **Flexibility in evidence types is more valuable than formal incentives:** What drives engagement isn't necessarily longer exclusivity but the ability to use conditional approvals or surrogate endpoints in high-UMN cases.
- **Desire for early regulatory dialogue and published guidance lists:** Sponsors want EMA to predefine which diseases/indications are UMNs or allow early validation of UMN claims, much like PIP templates.
- **UMNs should not be overly rigid or one-size-fits-all:** X emphasizes the need for case-by-case interpretation of UMNs to avoid exclusion of rare or emerging conditions.
- **HTA and regulatory divergence threatens alignment:** X highlights that HTA and regulatory perspectives on what constitutes an UMN or adequate evidence often diverge, especially regarding economic vs. clinical value.

External

- **Early engagement is essential, especially in UMN-based accelerated approvals:** Scientific advice early in development—especially when using a single pivotal trial—is critical.
- **Flexible trial designs are acceptable if evidence is strong:** Regulators don't require traditional designs if real-world or innovative methods provide robust evidence.
- **Claiming UMNs is not enough—context and quality of evidence matter:** Regulators independently assess if an UMN truly exists. Companies cannot just "tick the box."

- **Alignment between agencies (e.g., MHRA, EMA, FDA) is not guaranteed:** Even similar agencies act independently; entry into one expedited scheme doesn't ensure acceptance into another.

HTA / Market Access

Internal

- **HTA and regulatory standards differ—real-world value must be demonstrated:** Being approved does not mean the treatment will be reimbursed. HTAs expect proof of outcome relevance and health system benefit, not just regulatory compliance.
- **Comparator choice affects HTA strategy and post-approval positioning:** Choosing weak comparators can ease regulatory approval but hurt HTA evaluation later. Strategic alignment is needed across phases.
- **Flexible regulatory approaches must still ensure credible value claims:** While flexibility is welcome, especially for innovative therapies, weak evidence will not pass HTA scrutiny.
- **Cross-stakeholder UMN alignment would ease burden:** Different views on what counts as UMN across regulators, HTAs, and patients create inefficiencies and ambiguity. A harmonized framework is desired.

External

- Approval ≠ Reimbursement:
  HTAs may reject drugs that meet regulatory criteria if price is too high or clinical benefit unclear.
- **Need for harmonization and transparent UMN criteria:** (NEED) pushes for clearer, consistent definitions across EU to guide both industry and assessment bodies
- **Cost of data generation vs. need for robust standards:** Though expensive, industry must provide solid evidence on UMNs. HTA and regulatory systems should enforce data quality.

Patient Insights / RWE

Internal

- **Flexibility must serve meaningful outcomes, not shortcut standards:** Flexibility should enable faster, more efficient decisions without sacrificing scientific rigor.
- **Joint alignment between regulators and HTA is needed but difficult:** The fragmentation between EU member states creates inequalities and burdens for companies and patients.
- **Patient-centered endpoints often lack regulatory traction:** Regulators still favor "hard" endpoints despite patients valuing improvements in daily functioning or quality of life.
- **Early insights generation and cross-functional planning needed:** Involving patients and other functions early could improve both regulatory strategy and trial relevance.

5. Regulatory alignment

5.1. Impact of regulatory definitions on portfolio prioritization

Clinical

Internal

Non-contributors:

- Regulatory categories (e.g., UMN/HUMN) are seen as influencing value positioning more than trial design.
- Concern over exclusion of chronic burdensome conditions due to narrow UMN definitions.
- Clinical teams often work around regulatory constraints by tailoring endpoints to patient-specific symptoms, especially in rare diseases.
- Regulatory recognition (e.g., ODD, PRIME) may retroactively validate clinical direction, but initial prioritization relies on other factors.
- Regulatory uncertainty can delay or redirect prioritization in rare disease programs.

External

- Unclear UMN definitions create misalignment across regions.
- Clinical developers use regulatory incentives (e.g., fast-track) tactically but remain skeptical of their consistency.

Regulatory Affairs

Internal

- Regulatory definitions rarely drive portfolio decisions directly—business factors override.
- Misalignment between EMA and business priorities creates tension—e.g., avoiding pediatric obligations can kill EU launches.
- Regulatory decisions indirectly shape risk-benefit assessments and market expectations.
- Subpopulation focus is often disincentivized by regulators who expect broad impact, making prioritization riskier.
- Regional divergence in definitions (e.g., EU vs US) complicates global strategy.

External

- Statisticians play a proactive role early in regulatory engagement.
- Regulatory categorization should better reflect patient impact and practical feasibility.

HTA / Market Access

Internal

- Regulatory designations (e.g., UMN) don't guarantee HTA acceptance—real-world outcomes matter more.
- Narrow regulatory definitions risk excluding chronic and prevalent conditions from incentives.
- Early collaboration across departments helps align regulatory and value strategy.
- Fragmentation of definitions and incentives across countries (esp. in EU) challenges development planning.
- Incentives shape prioritization, but not always enough to drive development if market returns are uncertain.

External

- HTA definitions of unmet need require measurable health outcome improvements—not just process improvements.
- Incentives tied to orphan designation (e.g., added benefit assumption) distort HTA assessment.
- Efforts to align HTA and regulatory definitions (e.g., in Belgium) exist but face practical hurdles.

Patient Insights / RWE

Internal

- Regulatory and HTA alignment is ideal but unlikely due to fragmented systems.
- Lack of harmonized definitions across regions leads to patient access inequality.
- Early regulatory engagement (e.g., ODD) is now better integrated in RWE teams' planning.
- Practical RWE and patient data must inform regulatory claims to strengthen the case for UMNs.

5.2. Differences in requirements (EMA/FDA/MHRA - HTA). Divergences/similarities.

Clinical

Internal

- **Divergences in regulatory expectations:** FDA tends to be more flexible in accepting biomarkers and surrogate endpoints, especially in rare diseases, while EMA requires more structured, long-term, and comparative evidence.
- **EMA expectations include economic and comparative logic, anticipating HTA concerns more than FDA:** EMA demands evidence not just of safety/efficacy but also of superiority over existing treatments, reflecting early integration of economic reasoning.
- **Rare diseases allow more creative trial designs (especially in US):** FDA encourages creativity in evidence generation for rare indications; Europe is more cautious.
- **Early regulatory consultation is necessary to anticipate alignment gaps:** Internal teams systematically engage early with regulators (FDA/EMA) to flag feasibility or red flag concerns.

External

- **Labeling flexibility varies across regions:** Europe has stricter rules on what can be included in labels; US labels allow more flexibility in narrative.
- **Regulatory flexibility shapes development strategies:** FDA allows earlier engagements and faster timelines (e.g., rolling submissions), creating incentive to prioritize US development. [Expanded via transcript]
- **Strategic divergence between regulatory and HTA value demonstration:** HTA needs go beyond clean data to patient-relevant outcomes, while regulators still prioritize technical efficacy/safety.

Regulatory Affairs

Internal

- **EMA requires higher evidence standard (comparators, 3-arm studies):** EMA generally requires head-to-head comparisons or active comparator trials, unlike FDA where such designs are often optional.
- **FDA allows more leeway in evidence and may accept post-marketing commitments:** FDA may defer evidence generation (e.g., confirmatory trials), offering more flexibility in approval pathway.
- **EMA and FDA have diverging definitions of what qualifies as "high unmet need":** Different incentive triggers and designation rules (e.g., breakthrough vs. orphan) across agencies affect strategic planning.

External

- **MHRA and EMA are mostly aligned, but act independently:** Approval by EMA does not automatically translate to UK access; eligibility criteria for expedited pathways differ.
- **Statisticians assess evidence robustness—not UMN qualification:** At MHRA, unmet need status is determined clinically; evidence quality remains essential regardless of designation.
- **Rarity or UMN does not lower the evidentiary bar:** Even in rare diseases, claims of UMN do not lead to relaxed standards unless clearly justified.
- **FDA tends to accommodate early-phase flexibility:** Expedited programs in the US (e.g., breakthrough therapy) hinge on early evidence, even if full datasets aren't yet available.

HTA / Market Access

Internal

- **HTA assessments demand added value beyond regulatory success:** Approval based on surrogate or narrow endpoints is insufficient—HTAs require proof of real-world benefit, cost-offsets, and quality-of-life improvements.
- **Comparator selection is strategic for HTA positioning:** Choosing weak or outdated comparators may ease regulatory approval but weakens value arguments during HTA assessment, particularly in Germany or France.
- **Lack of alignment creates strategic tensions:** A drug may be approvable but not reimbursable; early HTA consideration is critical.
- **Joint HTA-regulator initiatives exist but fall short in impact:** EMA-HTA early advice is available but underused; country-level autonomy persists in pricing/access.

External

- **HTA requires comparative and measurable benefit over standard of care:** HTAs demand outcomes that show added value beyond placebo or broad "patient care" claims accepted by regulators.
- **Orphan designation misalignments persist:** Orphan status may secure regulatory incentives but doesn't guarantee HTA acceptance unless health outcomes improve.
- **HTA bodies operate independently despite JCA initiatives:** Even with joint clinical assessments, final reimbursement decisions remain national.
- **Lack of standardized patient-centric data weakens HTA submissions:** Despite growing awareness, companies often fail to plan for PRO and QoL data early enough.

Internal

- **Patient input is crucial but under-integrated into regulatory and HTA alignment:** Agencies increasingly value patient-relevant outcomes, yet these are often underused in early trial design.
- **Real-world evidence (RWE) is variably accepted:** FDA shows more openness than EMA/HTA to use RWE, especially in rare diseases; however, evidentiary standards must still be met. Expanded
- **Patient-reported outcomes are valuable but not consistently prioritized:** PROs are acknowledged as important, yet still seen as "supportive" rather than pivotal by some agencies. Expanded

5.3. Balancing regulatory clarity with flexibility to meet evolving medical needs

Clinical

Internal

- **Tension between regulatory clarity and real-world variability in rare diseases:** A rigid definition of UMNs risks excluding valid but atypical patient needs, especially in rare or heterogeneous diseases. Flexibility is crucial to allow for case-specific nuances
- **Patient relevance is hard to quantify but essential for alignment:** Clinical endpoints often fail to reflect what actually matters to patients (e.g., fatigue, daily activities), and regulatory clarity shouldn't ignore these lived experiences.
- **Regulatory granularity must be balanced with innovation:** Too much specificity in guidelines can undermine novel approaches and early-stage decisions. Clinical teams prefer adaptable standards that evolve with science.
- **Misalignment within companies on UMN implications:** Different internal teams prioritize regulatory alignment vs. real-world impact, which creates ambiguity in early-phase planning.

External

- **UMNs require early strategic framing but current definitions are vague:** The ambiguity around what counts as an UMN complicates early-stage clinical strategy and endpoint selection. A shared understanding would aid forward planning.
- **FDA's guidance lacks clarity on demonstrating alignment with UMNs post-designation:** Receiving a designation (e.g., BTD) does not ensure regulators will later agree the product addressed an UMN—more guidance is needed post-designation.
- **Flexibility must be scientifically justified:** Relaxed evidentiary standards should not be assumed; regulators demand robust data regardless of "UMN" status.

Regulatory Affairs

Internal

- **Flexibility in evidence types is more valuable than formal incentives:** What drives engagement isn't necessarily longer exclusivity but the ability to use conditional approvals or surrogate endpoints in high-UMN cases.
- **Desire for early regulatory dialogue and published guidance lists:** Sponsors want EMA to predefine which diseases/indications are UMNs or allow early validation of UMN claims, much like PIP templates. revised
- **UMNs should not be overly rigid or one-size-fits-all:** X emphasizes the need for case-by-case interpretation of UMNs to avoid exclusion of rare or emerging conditions.
- **HTA and regulatory divergence threatens alignment:** X highlights that HTA and regulatory perspectives on what constitutes an UMN or adequate evidence often diverge, especially regarding economic vs. clinical value.

External

- **Early engagement is essential, especially in UMN-based accelerated approvals:** Scientific advice early in development—especially when using a single pivotal trial—is critical.
- **Flexible trial designs are acceptable if evidence is strong:** Regulators don't require traditional designs if real-world or innovative methods provide robust evidence.
- **Claiming UMNs is not enough—context and quality of evidence matter:** Regulators independently assess if an UMN truly exists. Companies cannot just "tick the box."
- **Alignment between agencies (e.g., MHRA, EMA, FDA) is not guaranteed:** Even similar agencies act independently; entry into one expedited scheme doesn't ensure acceptance into another.

HTA / Market Access

Internal

- **HTA and regulatory standards differ—real-world value must be demonstrated:** Being approved does not mean the treatment will be reimbursed. HTAs expect proof of outcome relevance and health system benefit, not just regulatory compliance.

- **Comparator choice affects HTA strategy and post-approval positioning:** Choosing weak comparators can ease regulatory approval but hurt HTA evaluation later. Strategic alignment is needed across phases.
- **Flexible regulatory approaches must still ensure credible value claims:** While flexibility is welcome, especially for innovative therapies, weak evidence will not pass HTA scrutiny.
- **Cross-stakeholder UMN alignment would ease burden:** Different views on what counts as UMN across regulators, HTAs, and patients create inefficiencies and ambiguity. A harmonized framework is desired.

External

- Approval ≠ Reimbursement: HTAs may reject drugs that meet regulatory criteria if price is too high or clinical benefit unclear.
- **Need for harmonization and transparent UMN criteria:** (NEED) pushes for clearer, consistent definitions across EU to guide both industry and assessment bodies.
- **Cost of data generation vs. need for robust standards:** Though expensive, industry must provide solid evidence on UMNs. HTA and regulatory

Patient Insights / RWE

Internal

- **Flexibility must serve meaningful outcomes, not shortcut standards:** Flexibility should enable faster, more efficient decisions without sacrificing scientific rigor.
- **Joint alignment between regulators and HTA is needed but difficult:** The fragmentation between EU member states creates inequalities and burdens for companies and patients.
- **Patient-centered endpoints often lack regulatory traction:** Regulators still favor “hard” endpoints despite patients valuing improvements in daily functioning or quality of life. (completed with transcript)
- **Early insights generation and cross-functional planning needed:** Involving patients and other functions early could improve both regulatory strategy and trial relevance.

6. Proposed reforms and enhancements

6.1. Suggestions for improving regulatory frameworks and classification (Opinion)

Clinical

Internal

- **Harmonization between EMA and HTA is not working in practice:** Despite joint initiatives, national payer autonomy undermines the alignment goal. Developers still face fragmented frameworks.
- **Independent, multidisciplinary advisory boards can better define real patient needs:** Including external clinicians and patients would help identify what truly matters, beyond internal company views.
- **Definitions of ‘high’ unmet need remain ambiguous and subjective:** Unclear thresholds (e.g., morbidity/mortality) create inconsistency and uncertainty.
- **Internal misalignment between clinical and commercial views on unmet need:** Clinical relevance does not always match business goals, leading to strategic dissonance.
- **Rigid symptom-based endpoints can dilute actual need relevance:** Focusing narrowly on a symptom (e.g. muscle weakness) misses broader disease impact. Sometimes we just focus on other companies endpoints, just because they are doing it and we miss the basics.
- **Regulatory guidance should evolve but allow challenges when not clinically justified:** Start from existing guidelines but allow scientific questioning and flexibility.
- **Request for pediatric-style regulatory classification framework:** A pre-defined category system could support consistency and predictability in defining UMNs.

External

- **Non-drug interventions are often ignored in UMN evaluations:** Frameworks should consider efficacy of therapies like psychotherapy or devices.

- **Transparency in regulatory decisions would improve industry planning:** Publicly sharing UMN designations would help sponsors align development.
- **Push for structured regulatory guidance via advocacy groups:** External clinical stakeholders try to influence regulatory standards via societies or patient organizations.
- **Clearer, more useful guidance for patient engagement needed, especially for smaller companies:**
  Existing mechanisms are insufficiently standardized and underused.
- **Make UMN-linked designations harder to obtain, but more meaningful if granted:** Stricter criteria should be rewarded with tangible regulatory advantages.

Regulatory Affairs

Internal

- **Flexible, case-by-case interpretation of UMNs is preferred over strict definitions:** Each condition has unique context; rigid frameworks risk excluding valid cases.
- **Transparency from regulators on classification decisions remains limited:** There's still unpredictability on how EMA categorizes UMNs.
- **Joint EMA–payer advice is welcomed but currently underutilized and risky:** More structured engagement could help, but potential conflicts of interest remain.
- **Collaboration across authorities should aim to reduce duplication for sponsors:** Greater inter-agency alignment would reduce unnecessary regulatory burdens.
- **Understanding company and patient perspectives should be prioritized by regulators:** True impact of a disease can only be understood with more open dialogue.
- **Openness to small populations and rare needs is essential:** Regulatory systems should not reject innovations just due to small target populations.

External

- **Scientific rigor and methodological flexibility must coexist:** Stick to core scientific principles but avoid overly prescriptive frameworks.
- **Regulatory guidance is evolving but fragmented; robust evidence must remain central:** Scientific justification, not volume of data, should determine acceptability.
- **Lack of FDA consistency in recognizing non-drug therapies creates unfair gaps:** UMN frameworks should account for all effective treatments, not just pharmaceuticals.

HTA / Market Access

Internal

- **HTA requires more than regulatory success; real-world value must be shown:** Endpoints and regulatory standards alone are insufficient—evidence of impact is critical.
- **Incentives insufficient for targeting high-risk UMNs:** Reward structures (e.g., exclusivity, RDP) should be expanded to de-risk bold innovation.
- **Over-focusing on severe diseases risks neglecting chronic needs:** Prioritizing high mortality can freeze innovation in other conditions (e.g., asthma, psoriasis).
- **Comparator choice is strategic and affects HTA viability:** Using weak comparators may ease approval but leads to problems in value demonstration.
- **Endpoints must be quantitatively structured to justify value:** Weighted scoring systems (e.g. primary = 50%, etc.) help demonstrate improvement.
- **HTA frameworks lack the clear structure found in regulatory systems:** Companies must navigate less formalized and predictable HTA expectations.
- **X highlighted communication as a key tool in navigating HTA perception:** Small behavioral framing choices can influence how HTAs interpret value.

External

- **NEED project seen as leading model for system-level UMN identification:** Combines patient, burden, and data-driven methods within broader health policy goals.
- **Shift from industry-led to system-needs-driven innovation is crucial:** Innovation should respond to mapped needs, not vice versa.
- **Framework should cover prevention, devices, services—not just drugs:** A pharma-only lens limits applicability and overlooks systemic gaps.
- **Long-term sustainability of NEED requires political and financial backing:** Uptake across Europe hinges on institutional support.
- **Data gaps should be interpreted as UMN indicators themselves:** Lack of evidence highlights neglected areas needing attention.

Patient Insights / RWE

Internal

- **Early patient and RWE input improves alignment with true needs and endpoints:** Co-developing metrics ensures better strategic design and regulatory success.
- **Pre-competitive frameworks enable shared understanding of UMNs:** Joint early dialogue with experts and patients can define key needs objectively.
- **Current definitions are too narrow, exclude legitimate chronic or moderate conditions:** Equating unmet needs only with severe or orphan conditions is misleading.
- **Awareness of unmet needs is lacking even within companies:** Better internal communication and education on diseases is essential.
- **Bias toward rare/orphan diseases distorts resource allocation:** Highlighting orphan drugs may marginalize prevalent but under-addressed conditions.

6.2. Policy and incentive improvements to support therapies for UMNs

Clinical Development

Internal

- **HTA-regulatory misalignment complicates development planning** Early-phase development must address HTA and regulatory expectations simultaneously, increasing planning complexity.
- **EU incentives alone often insufficient to justify investment** European development is frequently pursued only due to existing U.S. development; otherwise, the business case fails.
- **Incentives are not only financial—reputation matters** Reputation-building, e.g., addressing difficult diseases, can be a motivator alongside financial incentives.
- **HTA practices differ across Europe, fragmenting strategy** Countries vary significantly in how they account for societal burden, making it difficult to generalize development strategies.
- **National definitions of unmet need reflect political and healthcare priorities** In some systems, broader societal burden matters; in others, cost takes precedence. [Expanded via transcript]
- **Incentives rarely discussed among junior clinical profiles** Early-career staff show limited awareness or engagement with regulatory incentives, suggesting strategic decisions occur at higher levels.

External

- **Designations (e.g., Breakthrough, Fast Track) enhance internal support** Even if offering modest regulatory acceleration, these designations help secure leadership backing and signal promise.
- **Resource gaps block early-phase development** Key studies (like natural history) may not be feasible without dedicated funding, regardless of unmet need.
- **Incentives act as symbolic validation tools** Beyond acceleration, these programs affirm that the project is addressing a relevant, high-priority problem.
- **Expedited pathways offer more reputational value than functional benefit** They often support funding and organizational momentum more than actual regulatory flexibility. [Expanded via transcript]

Regulatory Affairs

Internal

- **Incentives must offer more flexible approval options** Conditional approvals or acceptance of surrogate endpoints are more valuable than formal designations.
- **EMA perceived as rigid and slow compared to FDA** Gaining access to advice or designations is seen as burdensome at EMA, whereas FDA is more responsive and enabling.
- **High UMNs often face biological and technical barriers** Diseases with high UMN status are frequently very difficult to tackle from a development standpoint.
- **Calls for simpler, more predictable incentive pathways** There’s demand for mechanisms like the U.S. breakthrough designation that are easier to navigate. [Expanded via transcript]

External

- **Incentive awareness varies across agencies**
  While MHRA offers extended data protection for orphans, broader programs are less well known.
- **Expedited designations shape internal investment logic**
  They help companies justify allocation of resources and prioritize programs internally.
- **FDA's flexibility seen as development-enabling**
  The U.S. regulatory culture is viewed as more adaptable, especially under high unmet need. [Expanded via transcript]

HTA / Market Access

Internal

- **HTA expectations not aligned with regulatory criteria**
  Surrogate endpoints or narrow efficacy metrics that pass regulatory hurdles may fail in HTA due to lack of real-world or cost-related evidence.
- **Current incentives don't reward bold or high-risk development**
  Developing drugs for complex UMNs is not sufficiently compensated; exclusivity extensions or better pricing models could improve this.
- **Special funding mechanisms help offset HTA barriers**
  Innovative Medicines Fund (UK) and similar programs offer pathways for high-cost, non-cost-effective drugs.
- **Rigid methodologies harm innovative endpoints**
  HTA bodies prefer generic QoL tools over disease-specific ones, discouraging development aligned with patient needs.
- **Comparator selection is strategic in HTA**
  Choosing weak comparators may help with regulatory approval but weakens HTA arguments—especially in systems like Germany and France.
- **Ambitious trial designs should be better rewarded**
  Highly rigorous trials with strong endpoints still face financial uncertainty under current pricing systems.

External

- **Skepticism around new EU incentives: Don´t know if they are relevant enough to change the development programs.**
  6-month exclusivity extensions unlikely to change company behavior; broader access incentives would be more meaningful.
- **Ethical considerations gaining importance in defining UMNs**
  There's a growing push to incorporate ethical and equity concerns into HTA frameworks.
- **EU-wide access gaps undermine incentive value**
  New therapies are often not launched in Eastern Europe due to poor business cases, weakening the relevance of EU-level incentives.
- **Orphan drug incentives create imbalance**
  Current policies overly favor oncology, neglecting less profitable areas with equal or higher unmet need.

Patient Insights / RWE

Internal

- **Checklists prompted greater patient engagement**
  EMA documentation requirements led to increased industry inclusion of patients in earlier phases.
- **Patient insight teams do not directly influence incentives**
  Interviewees did not discuss policy incentives directly, indicating this topic lies outside their routine scope.

6.3. Early engagement with regulators and other stakeholders to facilitate drug development

Clinical

Internal

- **Internal alignment between clinical and commercial functions is necessary**
  Misalignment internally limits the effectiveness of external engagement.
- **Joint regulatory and HTA advice is conceptually valuable but adds complexity**
  While beneficial in theory, combined advice introduces operational challenges.
- **Clear regulatory definitions of UMNs are critical to avoid wasted investment**
  Unclear thresholds for UMNs result in high spend with uncertain ROI.
- **Skepticism about EU-level regulatory-payer coordination**
  Doubts about feasibility and harmonization across Europe.

- **Regulatory guidance should be questioned when misaligned with internal science**
  Companies should challenge misfit guidance constructively.
- **Listening to patients must avoid bias**
  Engagement needs to be genuine, not confirmatory.

External

- **Greater investment in early patient and prescriber engagement is needed**
  These voices improve relevance and legitimacy but are often underused.
- **Companies should look beyond regulatory checkboxes**
  Early engagement should shape strategy, not just meet formal milestones.
- **Regulatory agencies are more receptive than companies assume**
  Agencies like FDA are open to alternative paths, but only with early, transparent engagement.
- **Delays in engagement limit influence over regulatory design**
  Engaging too late means companies miss opportunities to shape requirements.
- **Early advice can clarify flexible paths or innovative endpoints**
  Early dialogue allows for negotiated use of non-standard data or approaches.

Regulatory Affairs

Internal

- **Scientific advice typically follows a national-to-EMA sequence**
  Stepwise engagement helps tailor designation and advice.
- **Payer advice is emerging but still uncommon**
  There is growing interest, but also concern about risks and unclear value.
- **Stakeholder workshops help regulators and industry align**
  These forums create mutual understanding.
- **Continuous dialogue supports adaptation**
  Ongoing contact with regulators enables response to emerging data.

External

- **Early advice is essential for limited evidence pathways**
  Especially for single pivotal studies or accelerated approvals.
  checked in transcript
- **Multiple mechanisms for early engagement are available**
  MHRA, EMA, and FDA offer structured and joint pathways.
  double checked with transcript
- **Strategic use of advice increases chances of success**
  Regulatory advice should be used to shape—not just validate—development plans.

HTA / Market Access

Internal

- **Early payer dialogue is key to de-risk development**
  Signals from payers on price or value can influence whether a program proceeds.
- **HTA advice should inform decisions before investment**
  Discussions on endpoints and comparators need to happen preclinically or at early design.
- **Global teams often fail to integrate HTA advice early enough**
  HTA expectations are overlooked in early-stage development.

External

- **UMN definitions must evolve to remain relevant**
  Static definitions limit regulatory and HTA flexibility.

Patient Insights / RWE

Internal

- **Early regulatory tools are underused**
  Mechanisms like ODD or protocol advice are not leveraged consistently.
- **Early regulatory planning should be integrated into strategy**
  Proactive use of designations and advice should be part of long-term planning.

7. What could companies do or change to address UMNs?

Clinical

Internal

- **RWE and payer perspectives should be included from the start of clinical development**
  Companies need to go beyond patient and medical input and incorporate real-world data and payer expectations early in trial planning.

- **Product planning documents (e.g., Product Concept plans) are underutilized for aligning on UMNs**
  These tools exist but are not widely known or used internally. Managers should revisit and communicate their implications regularly.
- **Awareness of existing documents and strategic opportunities needs to improve internally**
  There is often low visibility of cross-functional information and UMN-related opportunities. Annual reviews and better visibility across teams could help.
- **Lack of early-stage alignment between clinical development and unmet need strategy**
  Clinical teams often proceed based on trial design precedent or regulatory milestones rather than strategic UMN framing
- **Precedent-based thinking dominates clinical decisions**
  Some interviewees noted that trial design often follows past examples rather than tailoring to specific UMN contexts or stakeholder needs.

External

- **Regulatory arguments around UMNs can support alternative endpoints**
  By justifying unmet needs early, companies may push for inclusion of non-standard endpoints (e.g., patient-reported outcomes) in regulatory labels.
- **Patient and clinician views diverge; early engagement is needed to capture both**
  KOLs may not reflect real patient needs due to industry alignment—companies should independently validate patient-centered priorities.
- **Small companies lack guidance and resources to implement patient-focused engagement strategies**
  More structured support is needed for small- and mid-sized firms to replicate FDA-style patient involvement frameworks.

Regulatory Affairs

Internal

No relevant contributions identified

External

- **UMNs should be addressed from the early phase of development, not just in confirmatory stages**
  Early-stage development allows flexibility for innovation, including defining UMNs with patients and clinicians.
- **More structured engagement with patients would aid regulatory alignment**
  While the FDA hosts some patient-focused meetings, companies—especially smaller ones—lack the know-how to implement similar practices.
- **UMN framing can justify alternate regulatory endpoints**
  Identifying UMNs early can be leveraged to introduce meaningful but non-traditional endpoints during submissions.

HTA / Market Access

Internal

- **HTA assessments demand added value beyond regulatory success**
  Approval based on surrogate or narrow endpoints is insufficient—HTAs require proof of real-world benefit, cost-offsets, and quality-of-life improvements.
- **Comparator selection is strategic for HTA positioning**
  Choosing weak or outdated comparators may ease regulatory approval but weakens value arguments during HTA assessment, particularly in Germany or France.
- **External engagement is essential early on for designing relevant trials**
  HTA/payer, patient, and clinician input should be sought well before pivotal trial design.
- **Behavioral and perception framing matters in communicating value**
  Strategic language (e.g., "start" vs. "switch") and behavioral science influence prescriber and stakeholder adoption.

External

- **HTA and regulatory have different data needs—development must satisfy both**
  HTA assessments look at added benefit over standard of care, while regulators focus on benefit-risk; both should be planned for early.
- **Use of pragmatic randomized trials in real-world settings strengthens evidence for HTA**
  Registry-based trials (e.g., Nordic countries) offer more credible evidence than observational data.

- **Shift innovation model from supply-driven to needs-driven** Development should be based on identified UMNs, not the other way around.
- **Companies should adopt validated frameworks to define UMNs** Using consensus criteria (e.g., NEED project) and collecting relevant data from the start improves alignment.
- **Early dialogue with HTA bodies on UMN definitions is necessary** Clarifying what constitutes an unmet need and what evidence supports it should happen pre-trial.

Patient Insights / RWE

Internal

- **Low awareness of UMNs across the organization limits early identification** Internal education and communication about disease areas and patient needs are often lacking.
- **Early patient involvement is growing but was initially resisted internally** Patient engagement faced pushback but is now more accepted—partly due to positive outcomes in identifying endpoints.
- **Structured patient collaboration can improve trial relevance** Ongoing advisory boards or councils help ensure patient input is continuous and practical (e.g., identifying overlooked symptoms).
- **Gaps in internal experience from development to launch can hinder UMN planning** Some teams lack full-cycle knowledge, reducing foresight into what addressing UMNs effectively entails.
- **Limited integration of RWE expertise into decision-making** Internal RWE experts sometimes struggle to influence early decisions despite their relevance to UMN framing.
